# Surface-enhanced spectroscopy technology based on metamaterials

**DOI:** 10.1038/s41378-025-00905-7

**Published:** 2025-04-03

**Authors:** Dongxiao Li, Xueyuan Wu, Ziwei Chen, Tao Liu, Xiaojing Mu

**Affiliations:** Defense Key Disciplines Lab of Novel Micro-Nano Devices and System Technology, Key Laboratory of Optoelectronic Technology & Systems of Ministry of Education, International R & D center of Micro-nano Systems and New Materials Technology, Chongqing University, Chongqing, 400044 China

**Keywords:** Optical sensors, Nanophotonics and plasmonics, Structural properties

## Abstract

Surface-enhanced spectroscopy technology based on metamaterials has flourished in recent years, and the use of artificially designed subwavelength structures can effectively regulate light waves and electromagnetic fields, making it a valuable platform for sensing applications. With the continuous improvement of theory, several effective universal modes of metamaterials have gradually formed, including localized surface plasmon resonance (LSPR), Mie resonance, bound states in the continuum (BIC), and Fano resonance. This review begins by summarizing these core resonance mechanisms, followed by a comprehensive overview of six main surface-enhanced spectroscopy techniques across the electromagnetic spectrum: surface-enhanced fluorescence (SEF), surface-enhanced Raman scattering (SERS), surface-enhanced infrared absorption (SEIRA), terahertz (THz) sensing, refractive index (RI) sensing, and chiral sensing. These techniques cover a wide spectral range and address various optical characteristics, enabling the detection of molecular fingerprints, structural chirality, and refractive index changes. Additionally, this review summarized the combined use of different enhanced spectra, the integration with other advanced technologies, and the status of miniaturized metamaterial systems. Finally, we assess current challenges and future directions. Looking to the future, we anticipate that metamaterial-based surface-enhanced spectroscopy will play a transformative role in real-time, on-site detection across scientific, environmental, and biomedical fields.

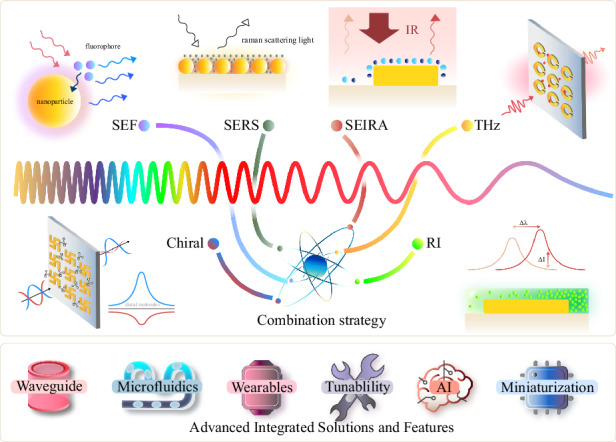

## Introduction

Metamaterials generally refer to materials with periodic structures designed and manufactured by humans, which often exhibit physical properties beyond conventional ones compared to natural materials, such as negative refractive index^1^, superlens effects^2^, and wave absorption^3,4^. At their core, metamaterials involve the artificial construction of material structures, enabling the creation of specific devices that manipulate the behavior of waves at a microscopic scale. Utilizing advanced micro- and nanoscale processes such as photolithography, nanoimprinting, and etching, a wide array of planar metamaterial devices can be mass-produced. This capability positions metamaterial-based planar devices as promising candidates for the development of integrated, multifunctional, micro-scale components with minimal losses in modern technology. After more than 20 years of vigorous development, it has expanded from its initial involvement in electromagnetism to the fields of optics^5,6^, mechanics^7^, thermodynamics^8,9^ and even acoustics^10,11^, showing a wide range of application scenarios not only in scientific research but also in the field of engineering. Specifically taking optics as an example, it can be designed for optical devices to control the propagation and manipulation of light, realizing functions such as optical stealth^12^, wavefront control^13,14^, superlensing, etc., and can also play a role in the fields of wireless communication^15,16^, imaging^17,18^ and sensing^19–21^.

Spectroscopic detection technology is a vital tool for analyzing the physical, chemical, and biological properties of substances. Its core principle involves probing the interaction between light and matter, allowing for the identification of target analytes’ composition and properties through absorption, emission, or scattering spectral information. This technology plays a crucial role in fields such as biomedicine^22,23^, analytical chemistry^24^, food safety^25,26^, and environmental monitoring^27^. For example, in biomedicine, spectroscopic techniques are widely used to detect biomolecules (such as proteins, nucleic acids, and carbohydrates) and their interactions, monitor disease biomarkers and pathogens, and even provide critical support in drug development and studies on cellular mechanisms. Despite the maturity and broad application of traditional spectroscopic techniques (such as infrared spectroscopy, Raman spectroscopy, and fluorescence spectroscopy), they still have certain limitations, such as limited sensitivity, high detection limits, inadequate resolution for complex samples, and the added cost and complexity of using labeled molecules for detection. In contrast, metamaterials offer a novel approach to enhancing spectroscopic detection (Table 1). By designing periodic structures, metamaterials can confine light to subwavelength scales and generate strong near-field electromagnetic enhancements. These enhanced hot spots significantly increase the interaction strength between light and analytes, extending the detection limits to lower concentrations. This performance leap makes metamaterial-based spectroscopic technologies a powerful, label-free, and non-destructive detection method, enabling efficient and rapid detection of trace samples. This also opens up new avenues for developing highly integrated, compact spectroscopic detection devices, with enormous potential in portable, high-sensitivity sensing and real-time detection applications.

**Table 1:** Comparison between traditional spectroscopic detection techniques and metamaterial-enhanced spectroscopic techniques

Parameter	Traditional spectroscopic detection techniques	Metamaterial-enhanced spectroscopic techniques
Detection sensitivity	Low, in micromolar to millimolar range	High, in nanomolar to picomolar range, single-molecule sensitivity
Limit of detection (LOD)	High, requires higher concentrations	Low, enhanced by localized field effects
Sample volume requirement	Large volumes (100 μL to 1 mL)	Small volumes (nano- to picoliter scale)
Spatial resolution	Micron-scale, limited by diffraction	Nanoscale, achieved through subwavelength structures
Device portability	Large, not portable	Miniaturized, suitable for portable, real-time detection
Cost and complexity	Low cost, mature but limited in miniaturization	Higher initial cost, but offers long-term value
Typical applications	Chemical analysis, biomolecular characterization, food safety, etc.	Trace molecule detection, biomarker diagnostics, environmental monitoring
Compatibility	Challenging to combine multiple technologies	Easily integrates multiple spectroscopic techniques on one chip

**Fig. 1:**
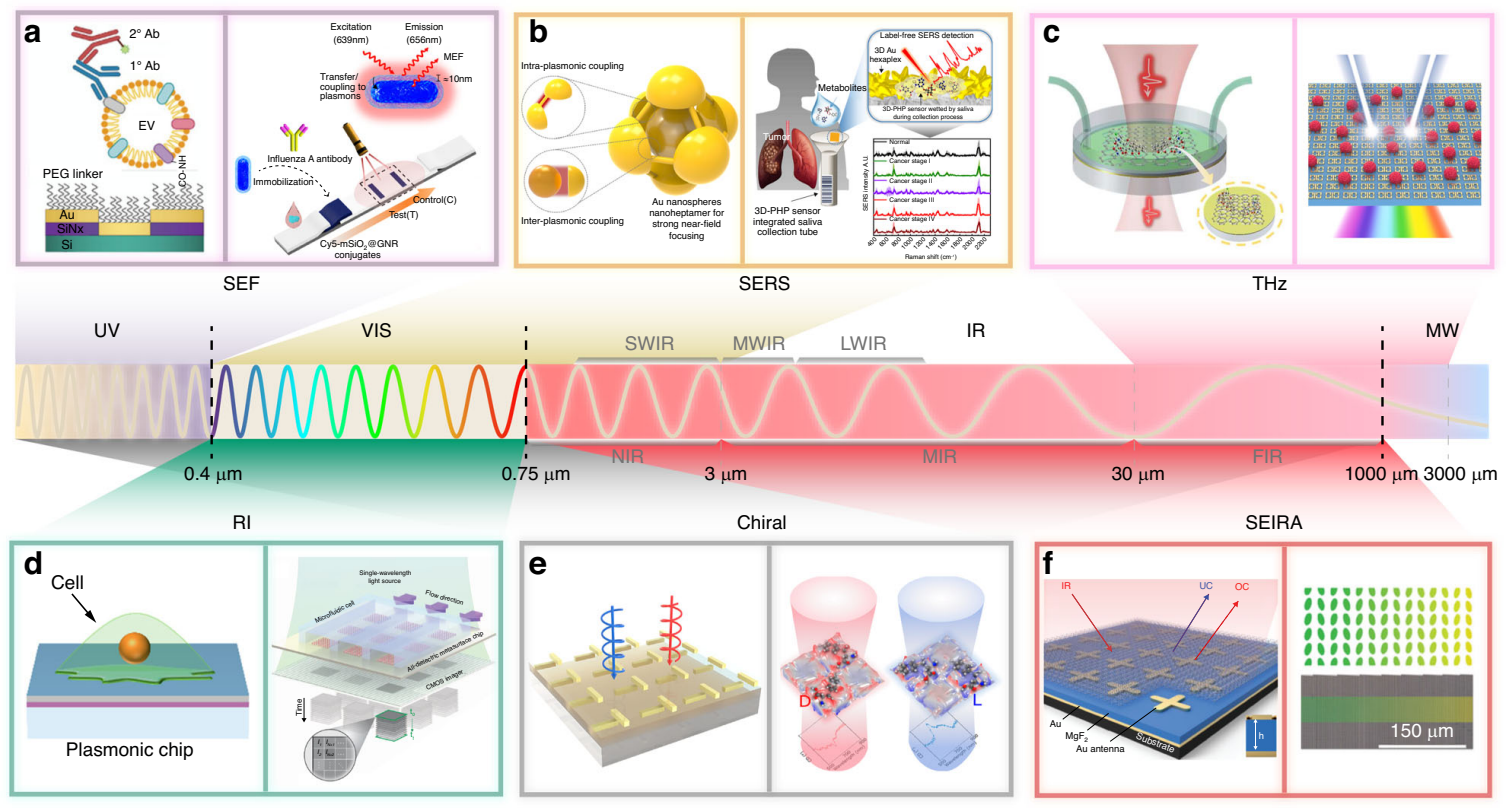
Metamaterials-based spectral enhancement from UV to THz optical bands. **a** Sensing applications of SEF spectroscopy. Left: A representative schematic of tumor-derived extracellular vesicles captured on the gold nanowell surface. Reproduced with permission^107^. Copyright 2023, Wiley. Right: Lateral-flow immunoassay sensor based on metal-enhanced fluorescence. Reproduced with permission^110^. Copyright 2023, American Chemical Society. **b** Substrates and application of SERS. Left: Au Octahedral Nanoheptamers for SERS. Reproduced with permission^143^. Copyright 2024, American Chemical Society. Right: Schematic illustration of the three-dimensional hexaplex-coated paper-based substrate sensor integrated with saliva collection tube for applications in human saliva sensing and lung cancer diagnosis. Reproduced with permission^154^. Copyright 2024, Elsevier. **c** Metamaterials-based THz sensing. Left: Metallic hole arrays-complementary asymmetry split ring metamaterial with graphene integrated into the microfluidic cell for sensing applications. Reproduced with permission^308^. Copyright 2021, Elsevier. Right: Terahertz meta-biosensor for lung cancer detection. Reproduced with permission^309^. Copyright 2022, Elsevier. **d** Metamaterials for refractive index sensing. Left: Periodic Aluminum nanohole arrays for measuring cell growth. Reproduced with permission^310^. Copyright 2021, American Chemical Society. Right: Optofluidic biosensors based on dielectric metamaterials for detecting extracellular vesicles by tracking the resonance wavelength or by the intensity change at a fixed probing wavelength. Reproduced with permission^125^. Copyright 2021, Springer Nature. **e** Metamaterials for chiral sensing. Left: design of Born-Kuhn type plasmonic nanodimers to achieve sensitive response to analytes corresponding to expected circular dichroism. Reproduced with permission^205^. Copyright 2023, American Chemical Society. Right: A chiral plasmonic sensor composed of a racemic mixture of gammadions with no intrinsic circular dichroism (CD). Reproduced with permission^197^. Copyright 2018, American Chemical Society. **f** Surface-enhanced infrared metamaterial. Left: Representative plasma nanoantennas. Reproduced with permission^173^. Copyright 2023, Wiley. Right: Gradient high-Q dielectric metamaterial that comprises pairs of tilted Ge ellipses. Reproduced with permission^183^. Copyright 2024, Wiley

Currently, reports related to spectral detection have become almost inseparable from metamaterials, and enhanced spectroscopy has fully covered the region of the electromagnetic spectrum that belongs to light waves. As shown in Fig. 1, we have systematically summarized six techniques involving metamaterials-based spectral enhancement from ultraviolet (UV) to terahertz (THz) optical bands, which are (i) surface-enhanced fluorescence (SEF) for probing the luminescence intensity of fluorescent molecules; (ii) surface-enhanced Raman scattering (SERS) for obtaining characteristic peaks corresponding to the different vibrational modes of molecules; (iii) surface-enhanced infrared absorption (SEIRA) for specific identification of molecular fingerprint information; (iv) THz sensing for exploring specific terahertz wave information, where refractive index changes are evident in this band and are mostly exploited; (v) refractive index (RI) sensing, which exploits the difference in dielectric constants of the surrounding medium for non-specific detection; and (vi) chiral sensing for qualitative and quantitative identification of enantiomers. Among them, (i–iv) is limited to specific wavelengths according to the frequency of excitation, absorption, or emission of light by the metamaterial or the target molecule, while (v–vi) has a considerable range of applications in the spectrum due to the special properties of matter. Since their boundaries may be somewhat blurred or overlap, Fig. 1 are not entirely accurate, but the positioning and relationship of these techniques can be better understood from the perspective of the entire spectrum. In practice, although the various surface enhancement techniques employ differentiated unit structures, their basic material types are consistent. The three most dominant material choices for metamaterials nowadays include plasma metamaterials which are formed by precious metals^28,29^, all-dielectric metamaterials^30^, and hybrid metamaterials by association of metals and dielectrics. The choice of different materials requires consideration of the trade-off between high enhancement and low loss in devices^31,32^. In addition to substrate materials, research on metamaterials mainly focuses on two areas. The first is performance optimization, which includes achieving lower detection limits, higher sensitivity, and enhanced detection of mixtures. The second is the development of complete systems, with an emphasis on miniaturization, multifunctionality, and cost reduction.

In brief, the aim of this review is to systematically summarize the existing metamaterials-based surface enhancement techniques across the spectral range and help researchers quickly understand the current state of development and applications of spectral enhancement. Firstly, we explain the physical properties of the generic modes such as localized surface plasmon resonance (LSPR), Mie resonance, and bound states in the continuum (BIC), and introduce the mechanisms of the various types of surface enhancement spectral techniques mentioned. Secondly, we review the recent advances in SEF, SERS, SERIA, THz sensing, RI sensing, and chiral sensing, which are mainly related to novel materials or structures, effective strategies and systems, and practical applications. We further collate existing metamaterials that employ binding strategies with a view to achieving the joint use of multispectral. It is particularly mentioned that metamaterial structures exhibit disruptive potential in achieving miniaturized sensing applications. Finally, we point out the problems that still need to be solved and look forward to the future direction of metamaterials-based surface enhancement spectroscopy. We expect that the summary and discussion of enhancement techniques across the entire spectral range will be mutually informative and inspirational in guiding the efficient design of devices and in grasping the overall development trend.

## Principle and mechanism

### Principles of common modes

The modulation of spectral information is achieved by carefully designing and arranging the parameters of the metamaterial unit structure artificially and controlling its interaction with light waves. In this process, a variety of optical resonance modes are generated, excited by specific configurations and exhibiting their own unique characteristics. Fortunately, some of these modes tend to be generic and widely used throughout the whole metamaterials field, demonstrating robust performance and even more practical design choices. Here, we will begin by providing a brief description of the physical principles of several common modes.

In 1902, Wood observed an abnormal phenomenon in the reflection spectrum of electromagnetic waves when they were incident upon a metal surface, which marked the earliest discovery of the surface plasmon resonance (SPR) phenomenon^33^. Over a wide frequency range, the optical properties of metals can be explained by a plasma model, where a gas of free electrons moves against a fixed background of positive ion cores. When an electromagnetic field is excited by external light, the electrons oscillate in response to the applied electromagnetic field. One of the electromagnetic excitations propagates at the interface between a dielectric and a conductor, defined as Surface plasmon polaritons (SPP). It is evanescently confined in the perpendicular direction but can propagate a certain distance along the interface. These electromagnetic surface waves arise via the coupling of the electromagnetic fields to oscillations of the conductor’s electron plasma^34^. It is important to note that matching between the wavevector of the SPP and that of the medium is a prerequisite for the excitation of the SPP. Therefore, specific excitation setups such as prisms, gratings, and waveguides are typically required.

In the case where the size of the metal nanostructure is smaller than the incident wavelength when a photon strikes its surface, many free valence electrons present in the metal move in response to the incident light, generating a collective oscillation known as the localized surface plasma (LSP). They are constrained by boundary conditions near subwavelength structures rather than propagating along the interface. The LSP leads to a large enhancement of the electric field near the surface of the particles, which is maximal at the surface and decreases rapidly with the increase in distance. The optical extinction of the particle hit a maximum at the plasma resonance frequency^35^. The resonance phenomenon produced by the LSP is called localized surface plasmon resonance (LSPR)^36^. Due to the strong interaction of the LSPR with the incident light, the effective extinction cross-section of the metal nanoparticles is enlarged, and thus they are also called nanoantennas. Efficient antennas are not limited to the shape of a sphere but can be tuned to obtain a large spectrally tunable cross-section and produce large local field enhancements by adjusting the structure, positional height, and transverse-to-longitudinal ratio^29^. In contrast to the SPR, LSPR are non-conducting and do not have to use devices to match wave vectors^37^. It is widely used in plasmonic metamaterials and is deeply researched.

The existence of Mie resonance in high refractive index dielectric nanostructures is an alternative approach to developing low-loss metamaterials with rich optical functions. Their optical resonances originate from displacement currents caused by bounded electron oscillations, reducing the nonradiative losses and heating of the nanoresonator. In 1908, Gustav Mie achieved a rigorous analytical solution for elastic scattering by a uniform dielectric sphere by solving Maxwell’s equations^38^, which detail the scattering of light by spherical particles. The scattered electromagnetic field has been written as an infinite series in the vector spherical harmonics, the electromagnetic normal modes of the spherical particle^39^. Thus, the scattered electric field is characterized by the electric and magnetic Mie coefficients derived from this expansion. The electromagnetic normal modes represent the distinct patterns of electromagnetic oscillation that a particle is capable of exhibiting under excitation by an applied electromagnetic field. These modes are contingent upon the particle’s dimensions, morphology, material attributes, and the frequency of the incident electromagnetic waves. In brief, its scattered electromagnetic field is represented as a sum of electric-type (TM) and magnetic-type (TE) spherical harmonics^30^. Under the excitation of plane waves, high refractive index particles produce the electric and magnetic field patterns of the corresponding four lowest-order electric and magnetic resonances, which are magnetic dipole (MD), electric dipole (ED), magnetic quadrupole (MQ), and electric quadrupole (EQ). Taking the MD resonance model as an example, at a fixed nanosphere diameter, it occurs at the smallest frequency of the incident wave compared to other resonances. A notable feature is the comparable contribution of MD and ED to the nanoparticle scattering cross-section. Their strong response at light frequencies offers opportunities for interacting not only with the electric component of a light field but also with its magnetic component^40^. Dielectric resonators have also been shown to provide strong magnetic resonance and strong hot spots in the electric near field simultaneously^41^. These Mie resonance-related resonance modes extend to non-spherical particles and show an increasing trend of interest in the field of nanophotonics.

Recently, a concept that is often used with dielectric metamaterials is the bound state in the continuum (BIC), and it is very effective for the design of resonators with high-quality factors. BIC was originally discovered in quantum mechanics and later identified as a general wave phenomenon. It lies inside the continuum but remains localized with no radiation. Typically, BIC couples to the extended waves and radiates. However, when a bound state of one symmetry class is embedded in the continuous spectrum of another symmetry class, their coupling is forbidden as long as the symmetry is preserved^42^. This is called symmetry-protected BIC. There is also an incidentally formed BIC due to the accidental vanishing of the coupling coefficients to the radiation waves via continuous tuning of one or several system parameters, like the Friedrich-Wintgen scenario^43^. In practice, BIC can be realized as quasi-BIC (QBIC) by generating leakage resonances through symmetry breaking^44^, which is widely used in metamaterials to generate high Q-factor Fano resonances^45,46^.

The fundamental lineshape of a resonance is generally described by the Lorentzian formula. In 1961, Ugo Fano discovered a new type of resonance that exhibits a distinctly asymmetric shape in contrast to a Lorentzian resonance and now bears his name^47^. The microscopic origin of the Fano resonance arises from the constructive and destructive interference of a narrow discrete resonance with a broad spectral line or continuum^48^. When two resonance states satisfying the above conditions are superimposed, a resonance line shape with asymmetric distribution is presented. Plasmonic materials and metamaterials can achieve Fano resonance without demanding conditions by artificially designing the structure and size of the nanoantenna, along with adjustment of damping loss of the nanoantenna and the coupling rate between the two resonance states. The sharp Fano resonance inherently sensitizes to changes in geometry or local environment, where small perturbations can induce dramatic resonance or lineshape shifts. This property renders Fano resonant particularly attractive for a range of applications.

In recent years, exceptional points (EPs) in open systems have been demonstrated to exhibit remarkable sensitivity. EPs are unique physical phenomena that occur in non-Hermitian systems^49^. Unlike traditional Hermitian systems, non-Hermitian systems typically describe the dynamics of open environments where gain and loss coexist. The eigenvalues of their Hamiltonians can be complex, with the real part representing the system’s energy and the imaginary part reflecting gain or loss. It is crucial to distinguish EPs from diabolic points (DPs). Typically, DPs occur in Hermitian systems under degeneracy, where only the eigenvalues merge while the corresponding eigenstates remain orthogonal^50^. In contrast, at an EP, both the eigenvalues and eigenvectors coalesce and degenerate^51^. This degeneration significantly alters the system’s energy configuration, resulting in dimensional reduction and topological tilting. The unique characteristics of EPs endow systems with remarkable physical properties. For instance, near EP, the system shows extremely high sensitivity to external disturbances. This sensitivity comes from the fact that the change of its eigenvalues has a square root dependence on the system parameters^52^. This square root dependence makes EP present higher sensitivity in low-concentration molecular sensing^53^. Moreover, EPs possess distinctive topological properties^54,55^. When system parameters encircle EPs, the eigenvalues and eigenvectors can exchange or follow complex trajectories. Recently, research on EPs has expanded to include multimodal non-Hermitian systems and topological photonics, driving advancements in non-Hermitian physics^56^. These studies have also opened new avenues for designing high-performance sensors^53^, linewidth enhanced lasers^57^, and asymmetric vector wavefront modulation device^58,59^.

Clearly, the resonant modes mentioned above do not exhaust all the resonance mechanisms used for spectral detection. Nevertheless, they are singled out due to their widespread application and significant efficacy in current research. In addition, modes such as electromagnetically induced transparency (EIT)^60^, electromagnetic induced absorption (EIA)^61^ and surface lattice resonance (SLR)^62^ are commonly utilized for light wave modulation by metamaterials. Through manipulation of the material, geometry, and arrangement of metamaterial units, the types and frequency range of the optical resonance can be effectively tuned to attain specific functionalities. These functionalities play a crucial role in analyzing the spectral characteristics of substances, facilitating the development of novel sensors, and enhancing the sensitivity and precision of spectroscopic analysis techniques.

### Mechanisms of surface-enhanced spectroscopy

**Fig. 2:**
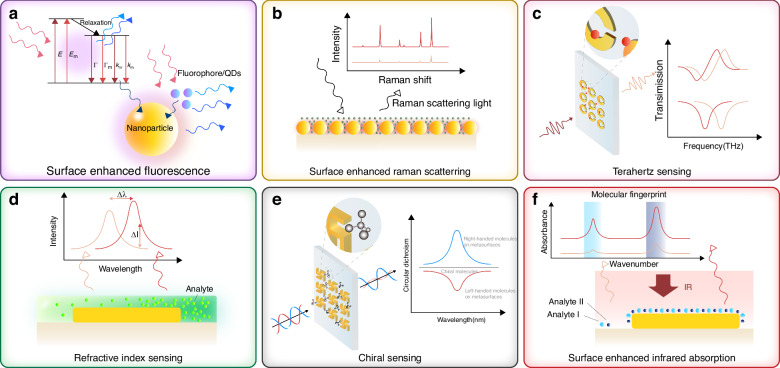
Schematic diagram of the mechanism of surface-enhanced spectroscopy. **a**–**f** Six major spectroscopic detection schemes are based on **a** SEF, **b** SERS, **c** THz sensing, **d** RI sensing, **e** chiral sensing, and **f** SEIRA

The previous section provided a brief introduction to the optical resonance modes commonly generated by metamaterials. This section will now focus on various enhanced spectroscopy techniques facilitated by metamaterials, elucidating their mechanisms for sensitive detection of the target analyte. Figure 2 shows a schematic representation of the mechanism of surface-enhanced spectroscopy, using the example of metallic nanostructured metamaterials.

Fluorescence, also known as photoluminescence, is a process that involves the absorption of photons followed by the emission of light. Generally, excited electrons undergo nonradiative transitions and release excess energy as heat. However, in fluorescence, a fraction of the excited electrons undergo radiative transitions and emit light with a longer wavelength than the absorbed light. Figure 2 illustrates the mechanism of achieving SEF in metal structures, and it can be attributed to three primary mechanisms. The most intuitive models attribute the observed spectral intensity enhancement to the local field enhancement associated with LSPR excitation in metallic nanostructures, which can increase the absorption and emission cross-sections of the sample. Both incident light and fluorescent molecules in the excited state may induce LSPR, and then the excitation intensity and efficiency of fluorescent molecules are improved^63^. SEF can only benefit from the local field enhancement of the incident field, so the enhancement factor (EF) can be calculated as ${{{\mathrm{EF}}}}_{{{\mathrm{SEF}}}}({\omega }_{{{\mathrm{ext}}}})={\left|E({\omega }_{{{\mathrm{ext}}}})/{E}_{0}({\omega }_{{{\mathrm{ext}}}})\right|}^{2}$, where $E$ and ${E}_{0}$ are the magnitudes of the electric field with and without the presence of plasmonic structures^64^, and *ω*_ext_ is the excitation frequency. Secondly, molecular emission is also affected by nearby metal nanoparticles altering the rate of molecular radiative decay. According to the Jablonski diagram^65^ (Fig. 2a, top left corner), the quantum yield (*Q*_*m*_) and lifetime (*τ*_*m*_) of the fluorophore near the surface are given by the following equations:

1 $${Q}_{m}=\frac{\Gamma +{\Gamma }_{m}}{\Gamma +{\Gamma }_{m}+{{\rm{k}}}_{{nr}}}$$

2 $${\tau }_{m}={(\Gamma +{\Gamma }_{m}+{{\rm{k}}}_{{nr}})}^{-1},$$

where Γ and k_nr_, respectively, represent the radiative decay rate and the nonradiative decay rate. Assuming the quenching effect of the metal surface is negligible (k_m_ = 0), the proximity of fluorescent dye to the metal particles accelerates the rate of radiative decay, with Γ_*m*_ representing the enhanced radiative decay rate. This results in a reduced fluorescence lifetime and an augmented fluorescence quantum yield^66^. Most importantly, according to the Förster-Dexter mechanism, when the excited state Fermi energy level of the adsorbed molecule is larger than that of the metal, the energy is rapidly transferred from the former to the latter. This energy transfer process can lead to two intertwined effects: fluorescence quenching due to nonradiative energy dissipation within the metal and fluorescence enhancement due to efficient far-field radiation^67^. Notably, when the distance between the fluorophore and the metal surface is optimal, fluorescence enhancement dominates^68^. This occurs because the coupling between the emission frequency of the fluorophore and the resonance of the metal particle enables the metal to radiate enhanced light at the same frequency through elastic scattering, amplifying the fluorescence signal. The three models’ interpretations of the enhanced fluorescence mechanism unfold from different perspectives but are fundamentally interrelated.

Compared to SEF, the mechanisms of SERS and SEIRA are more straightforward and mainly related to the near-field enhancement generated by metamaterials. Raman scattering is the inelastic photon scattering generated by the excitation of vibrational modes of a substance by light irradiation, including Stokes and anti-Stokes scattering. The frequency and relative intensity of Raman peaks reflect the type and strength of the corresponding atomic bonds. Figure 2b shows the mechanism of SERS, which includes contributions from electromagnetic enhancement and chemical enhancement. As the former is dominant, a strongly enhanced electric field increases the density of states of photons on the metal surface, which in turn increases the radiation rate of the scattering process and enhances the Raman scattering^69^. The Raman intensity enhancement is calculated through^70–72^

3 $${I}_{{{\mathrm{SERS}}}}\cong {I}_{0}{\left|\frac{E\left({\omega }_{{{\mathrm{ext}}}}\right)E\left({\omega }_{\det }\right)}{{E}_{0}\left({\omega }_{{{\mathrm{ext}}}}\right){E}_{0}\left({\omega }_{\det }\right)}\right|}^{2}$$

where *ω*_ext_ and *ω*_det_ are the excitation and detection frequencies, and Raman EF can be expressed as ${{{\mathrm{EF}}}}_{{{\mathrm{SERS}}}}={{\mathrm{EF}}}({\omega }_{{{\mathrm{ext}}}}){{\mathrm{EF}}}({\omega }_{\det })$, with ${{\mathrm{EF}}}(\omega )={\left|E(\omega )/{E}_{0}(\omega )\right|}^{2}$ ^69^. By changing the configuration of the nanostructures, the resonance frequency of the LSPR can be tuned to the ultraviolet, visible, or near-infrared wavelength to match the excitation and scattering wavelength for maximum enhancement^73,74^, so the size of the SERS substrate is limited to small nanoparticles. In the infrared band, different biomolecules exhibit unique vibrational fingerprint information due to differences in chemical bonds and functional groups, so that the structure and content of substances can be identified by infrared absorption spectroscopy. As shown in Fig. 2f, SEIRA can significantly enhance molecular IR vibrational signals, which is very effective in detecting trace molecular signals. Similar to SERS, both plasma-based electromagnetic effects and chemical effects related to adsorbed charge transfer contribute to the overall SEIRA enhancement^75^. Specifically, the electromagnetic effect is triggered by the generation of LSPR from metamaterials with significant near-field enhancement. Unlike the two-step enhancement mechanism in SERS, the enhanced absorption intensity in SEIRA is directly proportional to the local field enhancement of the incident light^72,76^. Therefore, the EF of SEIRA is defined as ${{{\mathrm{EF}}}}_{{{\mathrm{SEIRA}}}}(\omega )={\left|E({\omega }_{{{\mathrm{res}}}})/{E}_{0}({\omega }_{{{\mathrm{res}}}})\right|}^{2}$, where *ω*_res_ represents the resonance frequency. Molecules in the vicinity of the surface are affected by the generated electromagnetic field, leading to an increase in the vibrational dipole moment of the target molecule and a significant enhancement of the vibrational signal^77^. In addition, the interactions between plasma and molecular excitations can be understood through coupled harmonic oscillator models, temporally coupled-mode theory (TCMT), etc.^78^, which can also be a guide for better antenna design.

The terahertz (THz) wavelength range falls between 30 and 3000 µm (0.1–10 THz), bridging the gap between infrared and microwave regions. It occupies a crossover section of the electromagnetic spectrum between photonics and electronics, characterized by strong penetration, nonionizing properties, and sensitivity to weak interactions^79^. With the help of metamaterial, a strongly localized enhanced electromagnetic field can be generated at the device surface under the excitation of incident terahertz waves, and the mismatch between terahertz wavelength and biomolecular size was eliminated. Currently, the mechanism of refractive index change-induced frequency shift of THz wave resonance is the most utilized in sensing applications. As shown in Fig. 2c demonstrating a schematic of THz sensing, the Fano resonance excited by the classical asymmetric open-loop resonator supports a strong interaction of the terahertz wave with the analyte and undergoes a spectral frequency shift^80^. However, similar to infrared molecular fingerprint information, the rotational and vibrational transitions of molecules in the terahertz region also exhibit strong absorption and dispersion. The unique terahertz fingerprint spectrum presented is also information worth exploiting in the field of sensing. The fundamental principle of RI sensing is illustrated in Fig. 2d. As we previously discussed, the electromagnetic effects of metamaterials induce significant near-field enhancement. In the context of RI sensing portrayed here, this enhancement translates into a sensitive response to changes in the RI of the surrounding dielectric environment^81^. RI sensing, which allows for label-free and direct detection, is relatively mature compared to other enhancement techniques, and is a simple and effective strategy for sensing applications, mainly using wavelength or intensity variations to detect target analytes.

Chirality is widespread in nature and refers to mirror isomers of a substance or molecule that have spatial symmetry but cannot be recombined by operations such as translation and rotation. Many molecules in living organisms, such as amino acids, glucose, and ribonucleic acid, and drug molecules, such as the commonly used levofloxacin and dexibuprofen have chiral structures. Some disease patients contain characteristic chiral molecules in body fluid that show great differences compared with their healthy state, which opens the way for disease diagnosis using chiral properties. Most importantly, chiral molecules have unique optical rotation. When left-handed (LCP) and right-handed (RCP) circularly polarized light (CPL) pass through chiral molecules, they exhibit a significant difference in transmittance between the two polarization states^82^. This differential absorption, represented by the circular dichroism (CD) spectrum, makes spectral detection an effective tool for analyzing chiral molecules. Figure 2e demonstrates the typical mechanism of chiral sensing based on metamaterials to achieve enhanced chiral response in natural materials. The handedness of the enhanced electromagnetic field near the nanoparticle is governed by the chirality of the gammadion^83^. Despite the fact that the metamaterials exhibit chiral background spectra, the chiral coefficients of the adsorbed molecules are effectively enhanced^84^.

It can be seen that subtle variations exist among different metamaterial-enhanced spectroscopy techniques. However, fundamentally, they all rely on the construction of subwavelength structures within metamaterials, which generate robust near-field enhancement upon light excitation and effectively govern the interaction between light waves and molecules. This also implies that the multiple mechanisms are tightly interconnected. In the next section, we will present recent advances in each of the various technologies, and we believe that considering their intrinsic versatility and mobility during design studies can help to accelerate the progress of metamaterials in sensing, and also provide a wealth of resources and possibilities for innovation and application.

## Metamaterial platform enhanced spectroscopy for sensing

### Surface-enhanced fluorescence spectroscopy

Typically, ultraviolet (UV) light is used to excite the fluorescence of a substance, making it easy for humans to observe, and that is also the reason why we initially introduced surface-enhanced fluorescence spectroscopy. The enhancement of fluorescence spectral signals, as mentioned in the second section, results from the combined influence of multiple factors and can be explained by several different but interrelated theoretical models.

**Fig. 3:**
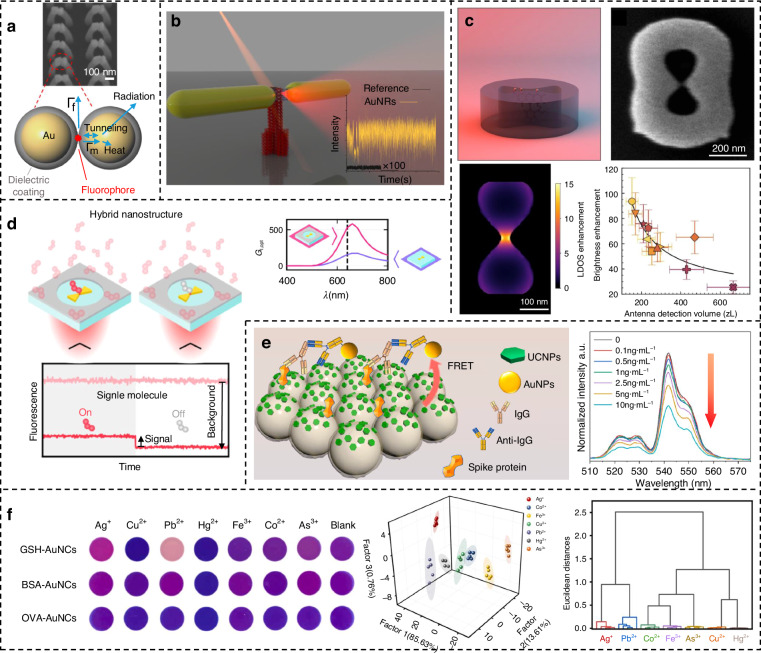
Various surface-enhanced fluorescence metamaterials. **a** The fluorophore molecules that are placed in plasmonic hot spots between pairs of collapsible nanofingers with tunable gap sizes at sub-nanometer precision. Reproduced with permission^87^. Copyright 2020, American Chemical Society. **b** Au NRs antenna dimers based on DNA origami technique. Reproduced with permission^90^. Copyright 2023, American Chemical Society. **c** GaP nanoantenna in infinity-shaped gallium. Reproduced with permission^93^. Copyright 2024, American Chemical Society. **d** An AiB composed of Au bow nanoantennas within an Al circular nanoaperture. Reproduced with permission^99^. Copyright 2023, American Chemical Society. **e** A FRET-based Novel Coronavirus IgG antibody sensor and its emission spectra of seven samples. Reproduced with permission^101^. Copyright 2023, Elsevier. **f** A fluorescence sensor array based on three gold nanoclusters for the direct identification and quantification of seven heavy metal ions with LDA and HCA. Reproduced with permission^113^. Copyright 2023, Elsevier

When it comes to enhanced surface platforms, it basically follows the common configuration of all spectral enhancement techniques. In pursuit of the advancement of SERS development, SEF has also rapidly gained momentum in recent years, demonstrating significant potential ranging from Single molecule detection to biosensing applications. Extending from the classic metal nanoparticle and nanoshell structure^63^, the enhanced substrate constantly evolving and upgrading. Iwanaga et al. achieved highly sensitive fluorescence detection of a wide range of targets, from antibodies to nucleic acids, on a metamaterials platform consisting of perforated silicon waveguides and stacked complementary gold nanostructures^85^. The enhancement around a single tip or nanoparticle (NP) is not as effective as the electric field in the gap between two NPs^86^. Song and his coworker introduced collapsible nanofingers with Au nanoparticles placed on top of those pairs in flexible polymers. Once capillary force is used to cause the fingers to collapse, the gap size between the Au NPs is determined by twice the thickness of the uniformly thin conformal dielectric layer deposited on them, enabling tunable sub-nanometer precision. In Fig. 3a, the fluorescent Nile blue molecules were easily trapped at the contact point, which is also the hottest spot exhibiting ultrastrong electromagnetic field enhancement^87^. Metal organic frameworks (MOFs) are increasingly used in the field of spectral enhancement^88^. Shen et al. integrated fluorescent probes into MOFs to design high-performance gas sensors selective for neurotoxin analogs^89^. Research has shown that Nanorods (NRs) outperform NPs for fluorescence enhancement. Through the DNA origami technique, which enabled the precise positioning of plasmonic NPs and fluorophores in self-assembled structures, Au NRs-based antenna dimers make fluorescence enhancement of commercial NIR dyes up to 1600-fold (Fig. 3b)^90^. Another paper, also using origami techniques, found that by tuning the spectral overlap between the intrinsic fluorescence of the dye and the nanoparticle resonance, it was essentially possible to design the emission spectrum of the system^91^. Metal plasmonic antennas always face the drawbacks of high optical loss and heat generation, while the corresponding resonant dielectric nanostructures have a unique low-loss resonant behavior that can offer a substitute for these metals, providing a flexible route to the manipulation of light at the nanoscale^92^. According to Fig. 3c, the study of gallium phosphide (GaP) nanoantenna in infinity-shaped structures reveals a significant average 63-fold fluorescence brightness enhancement, reaching a maximum of 93-fold for dye molecules within nanogaps ranging from 20 to 50 nm, as probed using fluorescence correlation spectroscopy (FCS)^93^. Another noteworthy study suggests that homogeneous, lossless, all-dielectric spheres with diameters in the mesoscale range between nano (100 nm) and micro (1 μm) scales, can offer surprisingly large fluorescence enhancements^94^. BIC-based metamaterials are rarely utilized in SEF, however, Bashiri et al. proposed a symmetry-breaking TiO_2_ structure realizing enhancement of the Eu^3+^ compound with directional control of different modes^95^.

In addition to common organic molecular fluorophores, semiconductor nanocrystals such as quantum dot (QD) photoluminescence have also been studied for fluorescence enhancement. It was demonstrated that the enhancement in quantum dots and plasma systems is associated with their charge exchange processes^96^. Furthermore, lanthanide ions also have photoluminescent properties and usually exhibit multiple emission lines. Zhang et al. integrated plasma and this kind of luminescence units into core-shell structures, and the selective emission enhancement of Eu^3+^ could be systematically modulated by controlling the size of the Au nanosphere core^97^. Despite the limited intensity achieved so far, the photoluminescence of metal nanoparticles can generate light signals from their own structures without any fluorescent dye. Kim al. designed and synthesized Au–Ag long-body nano snowman structures, facilitated by polysorbate 20, and enabled high and stable optical signals^98^. All these provide a variety of configuration possibilities for enhanced fluorescence technology.

One particular problem is that biosensing applications based on fluorescence detection often require single-molecule sensitivity in the presence of strong background signals. Antenna-in-box (AiB) platforms were discovered to achieve that at high fluorophore concentrations. Figure 3d illustrates an AiB composed of Au bow nanoantennas within an Al circular nanoaperture that improves background suppression while retaining the enhancement effect of gold^99^. Choi and Nam reported a new DNA nanoprobe based on the dual effects of target-specific plasmon-enhanced fluorescence and off-target plasmonic quenching, reducing background fluorescence of unhybridized DNA by introducing small gold nanoparticles as quenchers^100^. Among fluorescent nanomaterials, upconversion nanoparticles (UCNPs) can convert long-wavelength excitation light into short-wavelength emission. This unique property enables them to emit light under near-infrared excitation, effectively avoiding background noise. However, their fluorescence efficiency still needs urgent improvement. As shown in Fig. 3e, Hu et al. constructed a fluorescence resonant energy transfer (FRET) based Novel Coronavirus IgG antibody sensor^101^. Monolayer UCNPs as fluorescence energy donors were modified on the surface of the opal photonic crystal film, which could make the fluorescent signal more sensitive and Au NPs-modified Anti-IgG was adopted as the fluorescence energy receptor. With the increase of IgG concentration in the sample, the emission intensity of the detection area decreased gradually. The sensor showed remarkable detection performance for COVID-19 antibodies with the limit of detection (LOD) of 0.1 ng/mL. In addition, upconversion fluorescence has shown great potential in the field of imaging, e.g., enhancement of short-wave infrared upconversion emission of small organic dyes using gold nanorods (AuNRs) for in vivo imaging of ovarian cancer^102^. Another important challenge for the practical application of fluorescence strategies in the detection of biological samples is the immunity to interferences. Sun and his partners proposed a ratiometric fluorescent biosensing strategy for the sensitive detection of exosomal microRNA (miRNA), achieving a linear detection range of 0.1–50 pM and an LOD of 0.047 pM^103^. Incidentally, Zhang et al. developed a Förster resonance energy transfer (FRET)-based fluorescence biosensor to detect Alzheimer’s disease-associated miRNA^104^. This biosensor demonstrated one of the key advantages of fluorescence enhancement, achieving excellent response results both in vivo and in vitro with an LOD of 20.81 pM.

SEF spectroscopy facilitates early diagnosis and treatment of cancer, for example, it can detect model biomarkers of prostate cancer^105^. For the detection of matrix metalloproteinases, Au NRs-polymer substrates combined with dye pair decorated peptides enable ultrasensitive detection of their multiple phenotypes in multiple matrices down to 0.3 fg/mL^106^. Zhen et al. realized the detection of markers in extracellular vesicles of cholangiocarcinoma using gold nanowell structures^107^. The shell-isolated Au@MnO2 nanoparticle array also achieves a similar function^108^. SEF is also advantageous for other disease detection. The sub-pg/mL sensitivity of sepsis-associated cytokines was achieved within 1 h by nanoparticles spiked on gold nanodimple arrays and combined with tyramine chemical fluorescence signal amplification^109^. We must emphasize that rapidity and sensitivity are unparalleled advantages of surface spectral enhancement technology in disease diagnosis. Hong et al. proposed a metal fluorescent probe based on core-shell nanostructures using gold nanorod cores, mesoporous silica shells, and cyanine 5 fluorophore for optimization of a lateral-flow immunoassay platform. The influenza A virus nucleocapsid protein was tested with a limit of 0.52 pg/mL within 20 min^110^. Wang et al. demonstrated the enhancement of Lateral-flow assays by plasmonically active antibody-conjugated fluorescent gold nanorods. Detection of SARS-CoV-2 can be achieved in less than 20 min with high clinical specificity and sensitivity^111^. Also, Liu et al. used plasmonic-gold-enhanced near-infrared fluorescence to test its variants^112^. Although medical-related applications account for the largest use of surface-enhanced fluorescence, there are also opportunities in other areas of sensing. Meng et al. creatively proposed a fluorescent array sensor for the identification of multicomponent metal ion mixtures in complex systems at nM concentration level (Fig. 3f). Specifically, they used three gold nanoclusters with different ligands to quantitatively identify seven heavy metal ions from environmental waters, while the collected fluorescence fingerprints were processed by linear discriminant analysis (LDA) and hierarchical cluster analysis (HCA)^113^.

### Metamaterial refractive index sensing

The utilization of the RI of the analyte in enhanced spectral detection exists in all wavelength bands, however, in this subsection, the discussion mainly focuses on its use in the visible range and a few in the infrared range, which are both the most widely used range. In view of the RI change caused by the change of dielectric constant around the device, it has a promising application prospect in efficient and simple label-free detection.

**Fig. 4:**
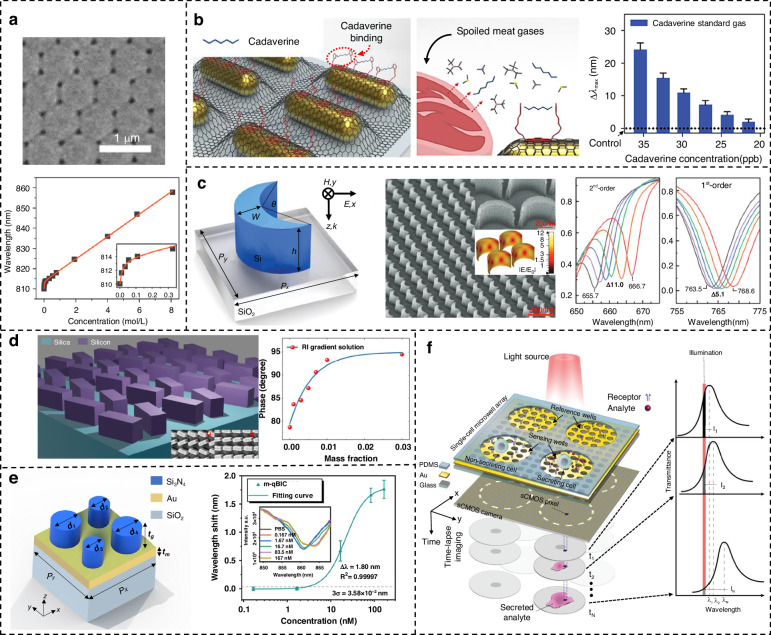
Different metamaterials for RI sensing. **a** Plasmonic sensors of a 2D hexagonal gold array and the interfacial RI obtained by fitting the experimentally acquired reflectance spectra. Reproduced with permission^114^. Copyright 2020, American Chemical Society. **b** A plasma gas sensor for the detection of cadaveric amines. Reproduced with permission^117^. Copyright 2023, American Chemical Society. **c** All-dielectric silicon crescent metamaterial and second-order resonance shifts. Reproduced with permission^119^. Copyright 2021, Wiley. **d** Dual-rectangular silicon pillar BIC metamaterial used for phase-interrogation RI sensing. Reproduced with permission^121^. Copyright 2023, American Chemical Society. **e** A hybrid cylindrical tetramer metamaterial which is based on two symmetry-protected qBIC modes. Reproduced with permission^123^. Copyright 2024, American Chemical Society. **f** Plasma microwell array system and the process of time-lapse plasmonic intensity images. Reproduced with permission^126^. Copyright 2023, Springer

The wavelength shifts of the resonance peak due to refractive index changes are non-linear over a wide range of analyte concentrations, especially at low concentrations where the sensitivity of the experimentally measured refractive index exceeds theoretical value. To provide a more reliable explanation of the experimental results with theoretical calculations, Zhan et al. used plasmonic sensors based on a 2D hexagonal gold array to measure glycerol solutions at various concentrations. The interfacial RI was quantitatively determined by disentangling the surface RI from the bulk RI, and the test results were in good agreement with the proposed theoretical (Fig. 4a)^114^.

Artificially tuned with design structures to achieve specific functions have always been the attraction of metamaterials. To take better advantage of this, the continuous exploration of useful structures in the quest for better results in devices cannot be neglected. Yu et al. fabricated an Au porous anodic alumina composite film with an inverted cone structure with Au nanoparticles and used it as a plasma substrate. Subsequently, the substrate is used to achieve the label-free detection of common reagents and glucose concentration with a sensitivity of 376 nm/RIU based on the refractive index^115^. Dong et al. proposed a metal-dielectric sandwich structure integrated with a grating-like subwavelength structure to achieve dual-region protein detection in the near and mid-infrared. More specifically, this metamaterial can potentially achieve protein recognition and trace detection with RI sensing realized in the near-infrared band^116^. The ultimate goal of adopting various means and designs is to expand the performance of sensors. It is worth considering trying external booted methods, such as applying additional energy, which may bring new perspectives to RI-based sensing applications. A plasma gas sensor for the detection of cadaveric amines has been reported to exhibit an ultrasensitive peak wavelength shift even in tiny molecules^117^. As shown in Fig. 4b, its substrate is covered with a single layer of graphene by chemical vapor deposition on gold nanorods and a chemical receptor immobilized on the graphene-coated nanorods to selectively trap the gas under test. By applying voltage to graphene, the detection limit is improved to 15.99 ppb, which can be used to detect meat spoilage. Li et al. fabricated complementary plasmonic nanopillar and plasmonic nanohole metamaterials, where the nanoholes exhibited higher sensitivity for the detection of tumor markers in clinical serum samples^118^.

All-dielectric metamaterials based on BIC can achieve ultra-high Q resonance, which is of great significance for improving the refractive index sensing performance. Wang et al. developed an all-dielectric silicon crescent metamaterial (Fig. 4c) driven by QBICs. Its fundamental and higher-order resonances can both be exploited for sensing ultrathin layers of biomolecules down to sub-nanomolar concentrations in air and buffer solutions, and the higher-order resonance allows bulk RI sensitivity to reach 326 nm/RIU^119^. Also based on QBIC all-dielectric surfaces, Watanabe and his coworkers succeeded in using silicon-on-quartz wafers to make metamaterials containing nanogaps, demonstrating ultra-high figure-of-merit (FOM)^120^. Remarkably, in addition to the electric quadrupole modes, which generate an external electric field, there are also magnetic dipole modes within the nanogap interacting with the surrounding dielectric. As shown in Fig. 4d, Liu et al. creatively proposed a phase-interrogation refractive index sensor made of a periodic array of two axially symmetrically distributed rectangular silicon columns on a quartz substrate, where the rotation angle represents the symmetry-breaking parameter. Combined with the microfluidic chip, the resonance wavelength shift of about 501 nm/RIU in solution detection is achieved, and the phase refractive index is about 2.7 × 10^4^ deg/RIU under TM polarization light source, which is comparable to the sensitivity of metal substrate^121^. A dual high-Q Fano resonances metamaterials was also reported^122^. Currently, the performance of metal-dielectric hybrid metamaterials is still under continuous validation. Luo et al. theoretically and experimentally demonstrated an optical sensor composed of a hybrid cylindrical tetramer metamaterial, shown in Fig. 4e. They achieved remarkable results, with a measured bulk sensitivity of 492.7 nm/RIU and an impressive FOM of 266.3 RIU^–1^ ^123^.

Given the excellent sensing performance demonstrated by RI-sensitive substrates, many researchers have turned their attention to systems with more complete functionality. RI-based detection biosensor systems are currently in the rapid development phase, showing promising progress toward the creation of more compact devices for rapid diagnostic applications. Altug’s group has done several works in building excellent and versatile sensing platforms. Initially, Yesilkoy et al. engineered all-dielectric asymmetric metamaterials supporting BIC that allow high-quality resonances, composed of arrays of metaunits with broken in-plane inversion symmetry^124^. They use an extremely narrow bandwidth and tunable excitation with high spectral resolution to build a hyperspectral data cube that contains full spectral information from the entire sensor array, where each sensor is mapped by complementary metal oxide semiconductor (CMOS) pixels. Later, Jahani and others proposed another signal acquisition and processing scheme using a similar sensitive substrate^125^. By configuring a single wavelength metamaterial chip into a large-area microarray and integrating it with microfluidics on an imaging platform, breast cancer extracellular vesicles containing exosomes are detected in real-time, enabling detection of, on average, 0.41 nanoparticle/µm^2^. This work not only employs suitable metamaterial but also provides a solution for constructing optical systems and signal outputs. Avoiding the use of bulky and complex spectrometers, they proposed a single-wavelength compact light source combined with an imaging camera and dynamically reconstructed the biomarker-induced spectral shift information using an optimal linear estimator. Recently, Ansaryan et al. reported on a new plasma microwell array system (Fig. 4f) that consists of four main parts: gold nanohole array substrate, PDMS micromesh, LED illumination source, and sCMOS camera^126^. These time-lapse plasmonic intensity images were collected at each position, followed by the process of corresponding intensity changes over time, and the binding event sensogram formed over the entire chip surface. This enables high-throughput spatiotemporal detection of multiple cell secretions.

### Surface-enhanced Raman scattering

Currently, SERS has developed into a promising detection and analysis technique. Traditional SERS substrates utilize noble metal NPs such as gold and silver to generate significant electromagnetic enhancement through surface plasmon resonance effects, achieving considerable enhancement factors. While these hot spots can provide significant signal enhancement, their formation is often stochastic and less controllable. In contrast, metamaterials are designed with precise control over their optical properties, enabling highly localized and tunable electromagnetic fields. This specificity can lead to more predictable and reproducible enhancements in spectroscopy compared to randomly arranged nanoparticles. It is crucial to determine the absolute enhancement values for quantitative analysis^127^, and an ideal nanoparticle array should be arranged periodically to provide uniform hot spots (Table 2)^128^. Thus, the combination of SERS and metamaterials also extends many novel performances.

**Table 2:** Comparison between traditional nanoparticles and metamaterials

Aspect	Traditional nanoparticles	With Metamaterials
Enhancement mechanism	Random hot spots from nanoparticle gaps	Near-electromagnetic fields
Control	Low (random arrangements)	High (precise design)
Reproducibility	Low (stochastic hot spots)	High (consistent enhancement)
Tunability	Limited (fixed by nanoparticle properties)	High (tailored for specific wavelengths)
Fabrication	Simple and cost-effective	Complex and expensive
Background noise	Higher (due to randomness)	Lower (controlled design)
Scalability	Easily scalable	Challenging to scale
Applications	Broad but less specialized	Specialized and high-precision applications

There are two commonly used strategies for constructing periodic SERS substrates: one is based on well-established nanofabrication techniques such as lithography and focused ion beam, which enable the adjustment of unit morphology in periodic SERS substrates^129^, and the other relies on the self-assembly of tunable nanoparticles to form periodic structures.

**Fig. 5:**
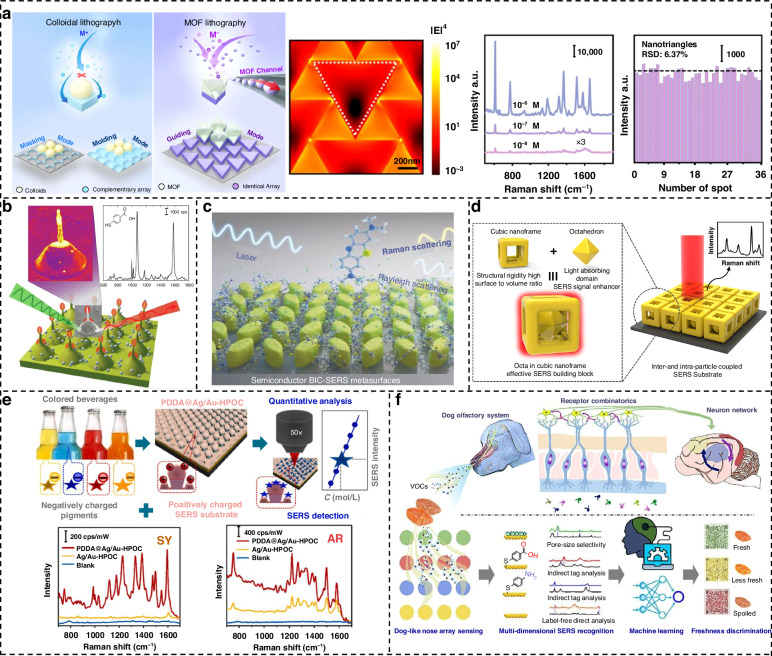
Various SERS based on metamaterials. **a** Two conventional colloidal lithography and MOF template-guided lithography. electromagnetic field distribution over the Ag nanotriangle array. SERS spectra of R6G molecules and SERS peak at 36 randomly chosen sites on it. Reproduced with permission^131^. Copyright 2023, Springer. **b** Jet-shaped SERS substrate and the enhanced scattering signal of 4-MBA. Reproduced with permission^134^. Copyright 2024, Elsevier. **c** BIC-driven TiO_2_ semiconductor SERS metamaterial and illustration of BIC-assisted absorption enhancement and spectral tunability. Reproduced with permission^141^. Copyright 2024, Wiley. **d** A solid Au octahedron is enclosed by an Au cubic nanoframe. Reproduced with permission^144^. Copyright 2024, American Chemical Society. **e** Quantitative SERS detection of dye molecules by PDDA@Ag/Au-HPOC substrate. SERS spectra of sunset yellow and Allura red. Reproduced with permission^148^. Copyright 2025, Elsevier. **f** An artificial nose of scalable plasmonic array gas sensor for Multi-Dimensional SERS recognition of volatile organic compounds. Reproduced with permission^149^. Copyright 2024, Elsevier

Xu et al. reported a highly controllable method to fabricate periodic bowtie SERS substrates with a narrow nanogap down to around 5 nm over a large area^130^. The plasmonic bowtie array is fabricated using holographic lithography and followed by depositions of Au and Ag, achieving an enhancement factor of 10^8^, which is 50 times higher than Au nanoparticle-assembled substrates. Lu et al. utilized assembled monolayer films of MOF microparticles as templates for metal electrodeposition, uncovering a previously unrecognized guided growth mechanism that enables the precise deposition of metallic films exclusively beneath the MOF microparticles^131^. As shown in Fig. 5a, in MOF template-guided lithography, electrodeposited metals grow precisely underneath the MOF microparticles, rendering the formation of metallic surface nanopatterns with a structure the same as that of the interface of the MOF template and the underneath substrate. Rhodamine 6 G (R6G) was used as a model molecule to evaluate the SERS performance of Ag nanotriangle arrays, detecting clear signals at concentrations as low as 10 nM. Typically, SERS measurements are conducted using a tightly focused laser with a finite spot diameter of ~1 μm. When such a small laser spot interacts with a large metallic plasmonic array, the array units out of the laser spot still dramatically influence the near-field enhancement in the spot and contribute to the SERS signal^132^. This finding is of great significance for guiding the design of efficient metal array structures. Recently, Li et al. used EBL to prepare different arrays of gold nanostructures, and used RhB as a probe molecule to systematically study the effect of different shapes of gold nanostructures on SERS response, elucidating the controllability of making uniform and stable SERS substrates^133^. Kernius et al. employed direct laser writing to fabricate periodic gold nanostructures with various shapes on the surface of a thin metal film^134^. Figure 5b presents a schematic of the jet-shaped SERS substrate and the enhanced scattering signal of 4-mercaptobenzoic acid (4-MBA) achieved by these nanostructures, resulting in an EF of 10^7^. This high EF underscores the potential of these arrays for future applications in single-molecule detection. In addition, Ye et al. introduced a method combining atomic force microscopy (AFM) scratching and nanoscaling to fabricate periodic folded Au nanostructures as SERS substrates^135^. These substrates exhibited excellent SERS performance, with p-aminothiophenol serving as a test molecule, achieving a detection limit as low as 10^−9^ M.

To realize uniform SERS measurements, the self-assembly method that can fabricate a close-packed arrangement of metal nanoparticles was adopted, generating spatially nearly uniform and dense hot spots on the substrate. Yao et al. developed a versatile strategy for the self-assembly of noble metal nanoparticles smaller than 15 nm into monolayer superlattices, demonstrating its applicability across a range of nanoparticle sizes and types^136^. Notably, the superlattice structure significantly enhances SERS performance, reducing the detection limit by 2 to 3 orders of magnitude compared to disordered films. Yoshiki et al. developed a nanostructure for which modal ultrastrong coupling is formed through densely packed AuNPs on Fabry–Pérot nanocavities using a self-assembly method^137^. Without taking precise control of the shape or spatial arrangement of the AuNPs on a nanoscale, the spatial variation in the signal intensity remained within 3% in SERS measurements using R6G under ultralow adsorption conditions (0.6 R6G molecules per AuNP). Tanwar et al. mentioned that the controlled assembly of large-scale arrays of bimetallic nanoparticles with uniformly distributed hot spots remains a challenge^138^. So, they presented a highly robust and reproducible method for creating large-area vertically aligned arrays of bimetallic Au-Ag nanorods, and it was demonstrated that the SERS optical performance of bimetallic nanorods was enhanced compared to their single metal counterparts.

SERS substrates based on metal oxide semiconductors, including Cu_2_O, ZnO, TiO_2_, etc., mainly rely on chemical enhancement. Although they sacrifice enhancement performance, they exhibit superior spectral stability and selectivity. To obtain high-performance SERS substrates, the strategy of utilizing both metals and semiconductors jointly has also attracted attention. For instance, Ag-decorated ZnFe₂O₄ nanotubes, grown on ordered Si pyramids using ZnO nanoneedles as sacrificial templates, are assembled into dandelion-like 3D aggregates with a periodic hexagonal close-packed arrangement^139^. This 3D-ordered SERS substrate can achieve the single molecule (10^−13^ M) detection level of R6G, with an EF of 3.14 × 10^9^. In a separate study, Gabinet and Osuji developed vertically oriented plasmonic nanorod arrays by hydrothermally synthesizing ZnO nanorods using block copolymer templates, followed by Au shell deposition. This structure enabled SERS detection of organic analytes at concentrations as low as 50 nM^140^. It is universally acknowledged that semiconductor SERS substrates typically rely on the PICT effect, but often suffer from extremely weak electromagnetic enhancement. An approach to improve the performance of semiconductor SERS substrates is to explore the use of BIC-driven metamaterials, amplifying the interaction between electromagnetic fields and light with matter. By showing high electric field enhancement (10^3^), a BIC-assisted semiconductor SERS metamaterial based on TiO_2_ was developed, achieving a detection sensitivity of 10^-8^ M on methylene blue in Fig. 5c^141^.

There have been numerous reports on the change in morphology of individual nanoparticles in the past^142^. A new effective strategy is to use Au nanoparticles as building blocks to construct spatial components. Zhao et al. reported a Synthesis method for Au nanoheptamers. This complex 3D NPs composed of six Au nanospheres located at the vertices of octahedral frames enclosing an inner Au nanosphere core at the center, all of which are connected by thin metal bridges, allowing multiple hot spots to be generated in the internal gap^143^. Another plasmonic particle-in-a-frame architecture in which a solid Au octahedron is enclosed by an Au cubic nanoframe emerges, shown in Fig. 5d^144^. Due to its unique structural features such as inner voids and ridges facilitating strong light–matter interactions, those nanoframe architecture represents a promising and complex plasmonic building block for SERS applications. As for the overall structure assembled from individual nanoparticles, Zhao et al. developed a method for large-scale patterning of superlattice structures^145^.

Currently, SERS technology is one of the most flourishing and promising technologies in the field of spectral sensing, with an ever-expanding array of novel concepts and groundbreaking configurations emerging to meet the demands of diverse industries and applications. Caligiuri et al. designed biodegradable and insoluble metastructures, featuring a square array of cellulose nanoholes coated with a thin silver layer, enabling the detection of 1,4-benzenedithiol molecules^146^. In the field of food safety, using self-assembly of C-Ag nanoparticles on nanoporous GaN for the Quantitative detection of thiram in real juice samples delivered a LOD of 10^−10^ M^147^. Zhang et al. prepared a novel PDDA@Ag/Au-HPOC substrate obtained by modifying an electropositive, functional poly (diallyldimethylammonium chloride) (PDDA) molecular layer on the surface of a periodic metal array substrate (Ag/Au-HPOC) using UV holographic lithography^148^. Notably, PDDA@Ag/Au-HPOC substrates enabled direct SERS detection of dyes in drink solutions without any pretreatment, offering a rapid and convenient method for dye molecule detection (Fig. 5e). It is worth mentioning that Qu et al. developed an interesting gas array sensor served as a dog-like artificial nose to offer a powerful SERS strategy for the food quality grading and bacterial contamination assessment. As shown in Fig. 5f, the gas array sensor included scalable plasmonic arrays with different surface functionalization and realized simultaneous analysis of four kinds of volatile organic compounds, i.e., bacterial metabolites, H_2_S, aldehyde, and biogenic amine, within the concentration range of 10^−1^–10^−8^ M^149^. Liu et al. developed a novel platform combining hyperbolic metamaterials (HMM) with bilayer Ag NPs to enhance SERS sensing^150^. The Ag NPs act as nanoantennas, converting Bloch surface plasmon polaritons into LSP, which amplify the electric field. The HMM further enhances the electric field within the structure, particularly at the interface between the Ag NPs and HMM. The platform exhibited strong SERS performance, achieving a LOD of 5.6 × 10^−^⁷ M for adenosine molecules and an EF of 4.4 × 10⁵. The HMM/bilayer Ag NPs structure exhibited a 205-fold enhancement in the electric field at the interparticle gap compared to monolayer Ag NPs. The metamaterial-based platform achieved high sensitivity and reproducibility in detecting adenosine molecules, highlighting its potential for practical SERS applications. Niu et al. developed an SU-8/Au/Al2O3/Au 3D micro-pole-array SERS substrate using SU-8 lithography and MDM layer deposition^151^. The substrate demonstrated high sensitivity with a LOD of 10⁻¹² mol/L for R6G. It was successfully applied to detect PCB-77 in soil with a detection limit of 10⁻⁸ mol/L, making it highly promising for environmental SERS sensing applications. Another notable study introduced a SERS sensor architecture based on Fe-ion-doped LN (FLN) crystals, which were modified with large-area laser-induced periodic surface structures (LIPSS) and subsequently decorated with Ag nanoparticles (Ag NPs)^152^. Leveraging the pyroelectric effect of the FLN crystal, the SERS signal was further enhanced during heating and cooling cycles. Interestingly, selective suppression of Raman signals was observed during cooling, broadening the application potential of the FLN/LIPSS/Ag substrate for detecting molecular complexes, such as crystal violet and 4-aminothiophenol pollutants in lake water.

In the realm of biomedicine, SERS presents a plethora of opportunities, particularly in its potential to revolutionize the landscape of disease diagnosis, offering precision, speed, and portability unparalleled by conventional methods. Luo et al. used a subwavelength plasmonic-gold nanohole array in an ultrathin gold film as the SERS substrate to identify the spectral features resulting from the modified cytosine in DNA. This label-free approach was further utilized to monitor the dynamic DNA epigenetic alterations in the TET protein-mediated oxidation process^153^. For cancer detection, SERS-based sensors also show great potential, such as hexaplex-shaped gold nanostructures for early-stage lung cancer screening^154^.

SERS analysis with machine learning is becoming an indispensable tool. Several works have been done to demonstrate that both standard machine learning techniques and convolutional neural networks can be effective in the quantification of SERS spectra^155^. The reported studies include the identification of mixed gaseous analyte^156^ and the determination of latent tuberculosis infection from plasma samples^157^. Fan’s group has conducted systematic research in this direction to address the challenges of rapid detection in complex sample systems. Initially, they used a simple principal component analysis (PCA) to rapidly differentiate between several bacteria under applied environmental stress^158^. Later, they took four kinds of silver nanoparticles to test a variety of tea samples, constructed multi-dimensional SERS profiles, and realized the differentiation of tea categories, origins, and grades with the help of PCA and linear discriminant analysis^159^. Further, multiple SERS fingerprints were combined with a one-dimensional convolutional neural network (1D-CNN) to achieve high accuracy in the identification and authentication of multiple agricultural products, including Flue-Cured Tobacco, Green Tea, and Purple Rice^160^. Recently, their SERS-1D-CNN platform has made progress in wastewater traceability by identifying wastewater content and plant information in multi-mixed samples with 97.33% identification accuracy^161^.

### Surface-enhanced infrared absorption spectroscopy

As a non-invasive, label-free detection tool, infrared spectroscopy is widely used in gas^162–165^, biomedicine^20,166–172^, and chemical substance detection^173–178^. Due to the mismatch between mid-infrared wavelength and nanometer molecular size, detection sensitivity is greatly reduced. The emergence of SEIRA has great potential to solve the problem of weak light–matter coupling ability. In particular, the emergence of plasmonic metamaterials can customize different resonance peaks through artificially designed subwavelength periodic structures according to target molecules and produce strong near-field enhancement, thereby improving the sensitivity of SEIRA^179^. As a complementary technology to SERS, infrared spectroscopy uses infrared light to measure the vibration and rotational motion of molecules in a sample and is more sensitive to different types of functional groups than Raman spectroscopy, providing more capabilities for multicomponent biochemical and gas detection^163,180^.

**Fig. 6:**
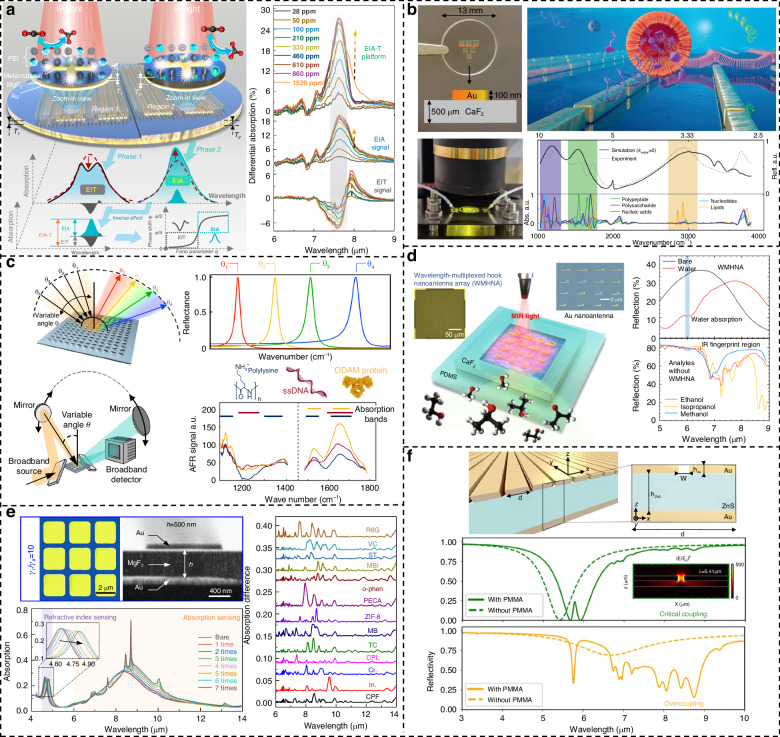
Various SEIRA based on metamaterials. **a** Fano-resonant dual-phase detection strategy. Corresponding difference absorption shows enhanced molecular signals. Reproduced with permission^164^. Copyright 2022, Wiley. **b** Multi-resonance metamaterials for detection of major biomolecules in water. Reproduced with permission^170^. Copyright 2021, Wiley. **c** Angle-multiplexed all-dielectric metasurfaces for broadband molecular fingerprint retrieval. Reproduced with permission^181^. Copyright 2019, AAAS. **d** Wavelength-multiplexing metamaterials for broadband-enhanced molecular fingerprinting. Reproduced with permission^175^. Copyright 2022, Springer. **e** Ultrasensitive molecular fingerprint retrieval using strongly detuned overcoupled plasmonic nanoantennas. Reproduced with permission^173^. Copyright 2023, Wiley. **f** Overcoupled resonator for broadband SEIRA. Reproduced with permission^182^. Copyright 2023, Springer

The traditional SEIRA platform lacks gas response time, selectivity, linearity, and sensitivity in gas detection research. In 2020, Zhou et al. began to combine metamaterial absorbers with MOFs, taking advantage of the large specific surface area of MOFs to increase the probability of combining plasmonic nanoantennas with gas molecules^19^. Fast response within 60 s for on-chip CH_4_ and CO_2_ is achieved, and both gases are detected within a wide concentration range of 0–25000 ppm. Later, Zhou et al. used loss engineering to construct a two-way strategy, using the low loss rate and high loss rate phases of Fano resonance to obtain the destructive and constructive signals of molecular vibrations, as shown in Fig. 6a^164^. When the two reverse signals are combined, the noise in the detection system is effectively suppressed, breaking through the limitations caused by noise and achieving a LOD of 13 ppm. In the same year, Zhou et al. also studied the gas adsorption capacity of polymer-MOF hybrid materials. They introduced amino groups into MOFs through post-synthetic modification to enhance the gas adsorption capacity of MOFs^165^. Together with the multi-hotspot nanoantenna strategy of nanogap coupling, ppb-level detection was achieved lower bound, providing new insights into the integration of advanced porous materials and nanophotonic devices.

Due to the non-invasive and label-free characteristics of SEIRA technology, it has shown unprecedented potential in detecting biological macromolecules. However, once the metal nanoantenna is designed and processed, its resonance peak is fixed and cannot match the vibration of complex biomolecule fingerprints. The resulting detuning greatly reduces the detection sensitivity. This problem was solved after researchers discovered the electrically tunable properties of graphene. Rodrigo et al. combined graphene with metamaterials and proposed a graphene-based tunable mid-infrared biosensor for label-free detection of protein monolayers^166^. The Fermi level of graphene is shifted by adjusting the external bias voltage, allowing the resonance peak generated by LSPR to be tuned. This enables the detection of the characteristic vibrational fingerprints of the protein molecule at 1660 and 1550 cm^−1^. In addition, graphene plasmons have stronger and tighter near-field intensity confinement than metal plasmons in the mid-infrared band. Subsequently, Hu et al. optimized a graphene-based mid-infrared biosensor to overcome the effects of plasmon-phonon coupling on graphene’s near-field strength and electrical tunability^167^. They proposed a broadband-response graphene-on-CaF_2_ platform, leveraging graphene’s broadband response and electrical tunability to broaden its biological detection applications and significantly enhance sensitivity. Multi-resonance metamaterials can also simultaneously amplify the vibration fingerprints of multiple biomolecules and have been widely developed and applied in the field of biosensing. In 2018, Rodrigo et al. introduced a multi-resonant metamaterial composed of self-similar overlapping nanoantenna arrays, achieving a 1000-fold near-field enhancement across multiple spectral bands^168^. This enabled simultaneous detection of characteristic absorption bands of lipids (2900 cm⁻¹) and peptides (1600 cm^−1^). The team later developed a three-resonance plasmonic metamaterial, as shown in Fig. 6b^170^. By integrating this metamaterial with microfluidics, they observed real-time vesicle capture, dual cargo release via perforation, and partial transition to planar lipid bilayers. Furthermore, incorporating machine learning for signal analysis allowed for more precise differentiation of multiple substances present in solutions.

Since the bandwidth of multi-resonant metamaterials with enhanced effects is limited, the interaction between light and matter significantly weakens when the molecular fingerprint vibration frequency does not match the plasmon resonance, reducing reusability. Developing plasmonic metamaterials with broadband responses can mitigate the spectral detuning issue to some extent. Researchers worldwide have proposed various methods to achieve broadband responses, each bringing unique insights to this challenge^169,173,175,176,181,182^. One approach combines discrete resonant peaks and achieves a meaningful broadband response through tuning. For instance, in 2018, Tittl et al. reported a mid-infrared nanophotonic sensor based on all-dielectric high-Q metamaterial elements, where each pixel has a high-Q narrowband resonance enabled by a BIC and subwavelength cavity modes^169^. The pixel array method divides the spectral range of 1350–1750 cm^−1^ into 10 × 10 resonance regions. The resonance frequency of each region is tuned by scaling the unit-cell lateral size factor S. This approach establishes a one-to-one correspondence between spatial position and wavelength, enabling molecular barcode imaging. In 2019, Leitis et al. developed a germanium-based metamaterial sensor using angle multiplexing (Fig. 6c)^181^. This design features sharp, surface-sensitive resonances. By adjusting the incident angle of mid-infrared light from 13° to 60°, over 200 resonant peaks are created, covering a broadband range of 1100 to 1800 cm^−1^. They demonstrated its potential by using DNA aptamers to detect human odontogenic ameloblast-associated protein (ODAM).

Another approach is to engineer a broadband response within a single resonance peak through loss optimization. In 2022, Ren et al. introduced a molecular identification platform using a wavelength-multiplexed hook nanoantenna array (WMHNA) (Fig. 6d)^175^. By optimizing the damping rate to reduce radiation losses, hook nanoantennas (HNAs) with gradient dimensions create a continuous ultra-broadband region (6–9 μm). They detected 15 absorption peaks of methanol, ethanol, isopropyl alcohol, and water using this array in combination with microfluidics. Machine learning algorithms further enhanced performance, achieving 100% classification accuracy with a 4:1 split between training and recognition sets. Recently, Richter et al. applied a similar resonance-gradient concept by adjusting unit-cell dimensions along the planar structure, creating continuous broadband coverage while maintaining a compact footprint^183^. In another study, Li et al. drew inspiration from temporal coupled-mode theory, using loss engineering to increase the bandwidth of overcoupled plasmonic nanoantennas (Fig. 6e)^173^. This approach can achieve high-intensity responses under spectral detuning without interference from asymmetric Fano line shapes, allowing direct readout of molecular signals. With machine learning, they achieved 100% identification accuracy of 13 biomolecules with highly detuned vibrational fingerprints. Furthermore, Li et al. further optimized the sensing performance of the overcoupled mode by reducing the gap between adjacent nanoantennas^184^. The optimized overcoupled resonator exhibited ultrasensitive (7.25% nm^−1^), ultrawideband (3–10 μm), and immune asymmetric Fano line shape sensing properties for polymethyl methacrylate (PMMA) molecules. Similarly, Paggi et al. designed an overcoupled resonator by adjusting the gap between nanoantennas (Fig. 6f)^182^. By loading PMMA molecules, they demonstrated that the overcoupled resonator improves sensitivity at lower Q-values, which defies conventional expectations. This method was validated by detecting dinitrotoluene (DNT). Both approaches represent significant advancements, providing novel methods and insights for SEIRA’s highly sensitive, versatile, and label-free applications.

### Metamaterial chiral sensing

The weak chirality of natural materials also leads to their small CD signals. Therefore, utilizing metamaterial structures to manipulate light waves for CD spectral enhancement remains a popular technical strategy. Chiral spectral sensing methodologies, resembling RI-dependent sensing, exhibit broad applicability across spectral ranges, in contrast to techniques reliant on specific wavelengths.

Based on the specific structural properties of antennas on metamaterials for chiral sensing, these materials can produce different responses to two types of circularly polarized light (CPL). Their unit cells can be classified as either chiral or achiral. Chiral unit cells typically employ complex shapes, such as a three-dimensional spiral structure^185^. In contrast, achiral unit cells achieve either a local chiral field or an overall chiral field by arranging individual achiral nanoantennas into an array^186,187^. It is important to note that some achiral antennas, like nanorods themselves, do not exhibit a CD signal, but the near-field interaction of the probed chiral material with these rods induces a difference in the absorption of CPL by the metal, which in turn exhibits an enhancement of the absorption signal and produced even higher enhancement factors than equivalent chiral structure^188^.

**Fig. 7:**
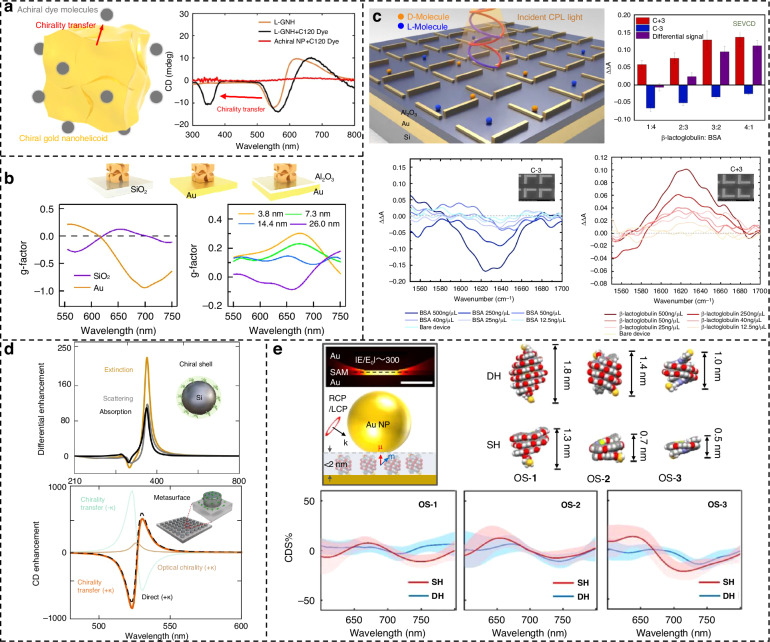
Different metamaterials for chiral sensing. **a** Schematic of an achiral dye@chiral gold nanohelicoid (GNH) hybrid system. Extinction CD spectra of l-GNHs, the hybrid of l-GNHs and C120, and the hybrid of achiral gold NP and C120, all in ethanol. Reproduced with permission^191^. Copyright 2024, American Chemical Society. **b** Scattering g-factor spectra of an L-handed chiral gold nanocube on SiO_2_ and Au substrate. Average g-factor spectra of the L-handed chiral gold nanocubes with different spacer thicknesses. Reproduced with permission^193^. Copyright 2023, American Chemical Society. **c** Schematic drawing of surface-enhanced vibrational CD platform. Extracted maximum signal difference from the CD spectrum. Experimental results of enhanced CD signal from BSA and β-lactoglobulin on C^−3^ and C^+3^ metamaterials, respectively. Reproduced with permission^194^. Copyright 2023, Springer. **d** A conformal chiral layer covers an array of silicon disk,s and chirality transfer can prevail over optical chirality in a dielectric metamaterial. Chirality transfer from a chiral shell in extinction, scattering, and absorption. Reproduced with permission^201^. Copyright 2023, American Chemical Society. **e** Schematic of Au NPoM nanostructure with helical SAM sandwiched in the nanogap. Configurations of double and single helices of OS-1, OS-2, and OS-3. Averaged CDS spectra of Au NPoM/OS in the state of DH and SH. Reproduced with permission^203^. Copyright 2024, Springer

To achieve chiral enhancement, it is natural to think at the outset of designing resonators with chiral nanoantennas and capable of generating chiral responses. Ceng et al. fabricate an air-suspended sandwiched chiral metamaterial consisting of two layers of metal nanostructures spaced by a silicon nitride membrane, where the chiral structure is composed of two blocks that are connected or separate^189^. In the experiment, the metamaterials not only demonstrated a sensitive response to the surrounding refractive index but also succeeded in distinguishing the chirality of the aspartic acid and propanediol. The local chiral hot spots generated by common local resonance modes such as LSPR and Mie resonances are susceptible to the interference of the chirality and concentration of analyte molecules. And the persistent inhomogeneity of the field enhancement is something that researchers want to address. Kim et al. present a conceptually new nanophotonic mode named as the collective CD of 2D helicoid crystals^190^. Arising from assembled isotropic, 432-symmetric chiral gold nanoparticles (helicoids), collective resonances (CRs) exhibit a strong and uniform chiral near field over a large volume above the 2D crystal plane. Their chirality sensor based on collective CD precisely quantifies handedness and concentration of proline and glucose through the empirical relations obtained in the experiment. Recently, a fascinating study has been done on the inverse process of chirality transfer, with similar metal nanostructures. By hybridizing achiral dye molecules with chiral gold nanohelicoids in solution (Fig. 7a), Chen et al. observe a chiral-plasmon-induced CD signal at the intrinsically achiral molecular absorption bands^191^. Another idea is to integrate a Pd shell on helicoid Au nanoparticles for hydrogen sensing. A significant circular dichroism red-shift as large as 206.1 nm can be achieved^192^. Figure 7b has also been shown that for these chiral gold nanocubes, optical chirality is inverted and enhanced when the substrate is changed from SiO_2_ to Au^193^.

When coming to the mid-infrared band, in addition to judging chirality from the wavelength shift, mid-IR vibrational CD spectroscopy also reveals vibrational information. Chen et al. proposed reflective chiral metamaterials consisting of two orthogonal resonant modes^194^. As shown in Fig. 7c, they detected two proteins and their mixtures, which were bovine serum albumin (BSA) and β-lactoglobulin, with similar vibrational modes and different optical chirality at the mid-IR wavelengths. The fundamental relationship between chiral molecules and planar chiral metamaterials is still worth exploring to help find the source of enhancement effects and better guide device design. Recently, they observed that strong near-field coupling induces less chiral response in chiral metamaterials, raising a presumption that near-field coupling strength can be an essential factor for far-field circular dichroism^195^. So, they proposed a quadruple-resonator system with a smaller chiral response and then verified the hypothesis experimentally. Their chiral metamaterial array for broadband sensing of glucose enantiomers achieved a detection limit of about 0.03 mM. Despite the relative complexity of the synthetic pathway, chiral MOFs are also used for enantiomer separations, chiral catalysis, and sensing. Kim et al. reported that zeolitic imidazolate framework (ZIF) can be grown on twisted bundles of cellulose nanocrystals^196^. And the templated chiral ZIF shows enantioselective recognition and chiral sensing abilities with a low limit of detection of 39 μM and the corresponding limit of chiral detection of 300 μM for representative chiral amino acid, D- and L- alanine.

The chiral antenna will produce a strong chiral background signal, which causes the limitation of sensing application to some extent. García-Guirado et al. proposed a chiral plasmonic sensor that enables the differentiation of enantiomers of phenylalanine^197^. It is composed of a racemic mixture of gammadions with no intrinsic CD, but high optical chirality and electric field enhancements in the near-fields. Also, researchers have attempted to attach chiral molecules to achiral nanostructures with a view to achieving greater enhancement of the CD spectrum. So they later employed an amorphous silicon dielectric cylindrical array of metamaterials, which are not inherently chiral, for probing Chiral phenylalanine molecules^198^. Ye et al. simulated a dielectric metamaterial consisting of achiral germanium (Ge) tetramer nanoresonators, which provides an accessible and high optical chirality enhancement in the mid-infrared region^199^. It relies heavily on the Mie resonance of the Ge resonator for strong magnetic and electric field enhancement, and its detection of chiral biospheres in the central region of the unit enables 183 C_E_. Holey dielectric disks are reported to achieve CD spectral enhancement in the detection of thin layers of chiral analytes^200^. Like nanorods, they are inherently achiral without any background signal. Mohammadi and his coworkers later explained the enhancement of circular dichroism in terms of a chirality transfer mechanism and found that it prevailed over optical chirality in the dielectric metamaterial-enhanced CD spectrum^201^. Moreover, it is illustrated from theoretical analyses in Fig. 7d that the ideal Mie resonator based on chirality transfer is a chiral shell-achiral nanosphere at long wavelengths and small radii. Achieving complex chiral responses through adjustable, simple designs is what we aim to observe. Gryb et al. demonstrated a geometrically simplest design of a two-dimensional planar chiral dielectric metamaterial^202^. The design avoids complex structures by simply arranging amorphous silicon rectangular rods on a glass plate and setting the rotation angle to produce chiral, as long as the rotation does not allow for out-of-plane mirror symmetries. It can be seen that the current research on chiral enhancement by non-chiral antennas is still limited. Many of them are only carried out to the stage of the study of the metamaterial itself, and the understanding of the specific enhancement mechanism from the principle level is not thorough enough. However, considering the simplicity of the preparation of non-chiral units, combined with the existing sensing examples, it is expected that this method will be better utilized in the future.

The limits of plasmon-enhanced chiral sensing are being pushed to the single-molecule level. In Fig. 7e, Zhang et al. designed a system made of chiral helical oligoamide sequences (OS) and achiral nanoparticle-on-mirror (NPoM) resonator, which enhances the chiral sensitivity in the quantum tunneling regime^203^. Currently, this system can detect a minimum of four molecules per single-Au particle characterized by circular differential scattering (CDS) spectroscopy. Incidentally, the chiral absorption features of most small molecules are in the UV band, and the use of UV-transparent materials such as diamond^204^ and AIN can be considered to design metamaterials with strong optical chirality in this band. It’s also worth mentioning that inverse design and algorithmic assistance can be considered universal tools for the development and optimization of plasma chiral sensors^205^.

In the field of selective recognition of enantiomers, there is a significant gap in the sensitivity of using metamaterials to enhance CD spectra compared to chemiluminescence^206^ methods, so further enhancement is necessary. Overall, chiral metamaterials are largely unexplored in their coupling with molecular.

### Metamaterial terahertz sensing

In recent years, due to rapid advancements in terahertz source and detection technologies, terahertz waves have garnered significant attention in fields such as wireless communications, chemistry, and biomedical sciences^79,80,207^. Artificially designed periodic structures in metamaterials have emerged as powerful tools for manipulating and enhancing THz waves^208,209^. Considering the special position of the THz band in the electromagnetic spectrum, this section will focus on the recent developments and applications of THz sensing individually.

In the design of metamaterials, including THz sensing, there is a common and effective strategy to achieve better performance and complex functions, which is adjusting the shape, size, and material of the structure. The most commonly used structure is the open resonance ring, from which many variants have arisen, such as the floating open ring^210^. Xu et al. proposed a dual-torus toroidal flexible metamaterial containing an array of overlapped split ring resonators. Microfluidic integrated dual-torus toroidal resonance experimentally shows a significantly higher sensitivity of 124.3 GHz/RIU compared to a single torus toroidal^211^. Qu et al. reported a quadruple rotation structure unit consisting of four identical metal open square rings and a nested metal square ring of the same size, featuring high sensitivity and high Q and FOM values^212^. In addition, there are special designs such as breaking Chinese Taichi-like rings^213^. By periodically arranging the unit cell which is a square gold ring with four T-shaped strips on a silicon substrate, a dual-band metamaterial absorber with resonant frequencies of 0.89 and 1.36 THz is proposed to achieve RI sensing with a sensitivity of 37 GHz RIU^-1^and exhibits perfect absorption characteristics with high Q-factor on both bands^214^. There are also multi-narrowband terahertz metamaterial absorbers such as patterned graphene^215^ and Au doughnut boxes^216^ on gold reflectors. Initially, scholars tend to select metals like gold and silver to construct metamaterial owing to their robust resonant effects. Then it gradually expanded to encompass the use of all-dielectric materials, exploring new possibilities for functionalization. H-shaped all-silicon arrays were reported to demonstrate tunable ultra-broadband terahertz wave absorption, revealing near unity absorption at 1 THz^217^. Using split half-cylinder silicon metamaterials combined with functionalized gold NPs allowed specific recognition of hemagglutinin tag protein^218^. It is increasingly common to take graphene into consideration because it may cause metamaterial electrically tunable over an extremely large broadband spectrum. Epstein et al. achieved this by combining nanosilver cubes with graphene^219^. THz biosensors for detecting casein molecules based on the hybridization of the metamaterial with graphitic carbon nitride, graphene, and heterojunction also show good performance^220^. Liu et al. proposed a dual-tunable metamaterial absorption modulator based on a hybrid vanadium dioxide-graphene configuration. The state of vanadium dioxide determines whether the absorber is wideband or narrowband and by controlling the Fermi level of graphene the absorptivity and resonant frequency can be modified^221^. The use of unique material properties results in a switchable terahertz absorber. Another novel terahertz metamaterial by etching subwavelength arrays on the surface of the single-walled carbon nanotube film was reported. In the detection of different concentrations of 2,4-dichlorophenoxyacetic acid solutions experiments, it is observed that the THz transmission peak magnitude of the device decreases from 1 to 0.79 at the resonance frequency of 1.38 THz when the concentration of 2,4-D solution increases from 0 ppm to 8 ppm^222^. It is indicated that all-dielectric materials with low intrinsic absorption and high-quality factors are powerful substitutes for metal materials.

Although chiral metamaterials can generate strong chiral optical responses, their Q factors remain low due to significant radiative and nonradiative losses. To address this issue, a special state of BIC has been proposed, which can confine light within the radiative continuum for an infinitely long time^223,224^. In physical systems, BIC manifests as QBIC with finite but still very high Q factors, offering unique light confinement and radiation characteristics. These properties have been successfully utilized to enhance the performance of nanophotonic devices, including twisted optical^225^, extraordinary optical forces^226^, and biochemical sensing^227^. For instance, by incorporating transverse magnetic and transverse electric QBIC modes in photonic crystal slabs (PCS), strong superchiral fields can be generated^228^. Such intense chiral fields provide opportunities for advancing chiral sensing applications^229,230^. When intrinsic chirality is introduced into BIC structures, chiral BICs with high Q factors and strong CD can be achieved. However, achieving chiral BICs requires breaking all mirror symmetries of the structure^231,232^. To this end, Zhang et al. introduced a second asymmetry parameter, the tilt angle α, into a classical dual-ellipse BIC design to further break the out-of-plane C2 symmetry^233^. Their research showed that the tilt angle α precisely manipulates the position of the C-point in momentum space, enabling QBIC with intrinsic chirality. Similarly, Chen et al. designed tilted geometric structures that broke both in-plane and out-of-plane symmetries, observing intrinsic chiral BICs in the continuum with CD values approaching 0.93 and Q factors exceeding 2663^234^. QBICs with intrinsic chirality offer new insights for the study of chiral light sources and detectors. However, the sensitivity of the C-point to geometric structures inevitably affects the Q factor. To robustly generate intrinsic C-points with high Q factors, Lv et al. proposed magneto-optical (MO) BICs in symmetric PCS^235^. These C-points exhibit robustness against structural geometric variations due to MO coupling in non-zero external magnetic fields. It is important to note that QBICs with intrinsic chirality are still at the forefront of research, and exploring their potential for chiral molecular sensing will be an exciting area of research.

**Fig. 8:**
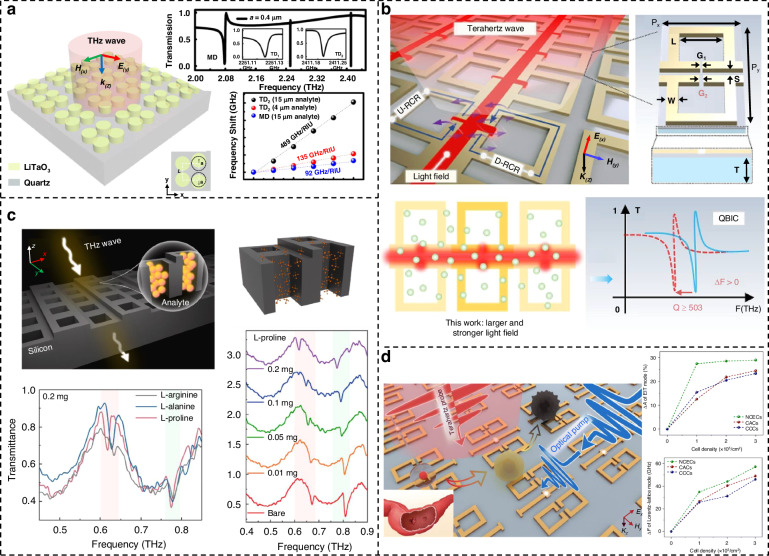
Different metamaterials for THz sensing. **a** A THz all-dielectric metamaterial with high-Q toroidal dipole resonance governed by BIC. Reproduced with permission^236^. Copyright 2021, Walter de Gruyter Gmbh. **b** QBIC metallic metamaterial biosensor based on wide-area and intense light–matter interaction. Reproduced with permission^237^. Copyright 2023, Elsevier. **c** An all-silicon THz metamaterial enables a passive trap of analyte. Reproduced with permission^238^. Copyright 2024, Elsevier. **d** THz metamaterials for ultrafast switchable sensing of colorectal cells. Reproduced with permission^242^. Copyright 2022, Royal Society of Chemistry

In recent years, researchers intend to realize High Q-factor resonance, which represents having the ability to detect small frequency shifts caused by trace analytes. One particular method is based on the BIC. In practice, BIC is an ideal situation but can be realized as QBIC by creating a leaky resonance through symmetry breaking. An all-dielectric metamaterial structure based on toroidal dipole (TD) resonance driven by BIC was proposed at THz frequencies. As shown in Fig. 8a, the unit cell consists of tetramer clusters, each of which is a cylinder deposited on the quartz substrate. The asymmetric parameter *a* means the introduce symmetry breaking that excites two TD modes. When cluster structure remains symmetric (a = 0 μm), only an easily excitable magnetic dipole resonance can be observed at 2.08 THz in the transmission spectrum. When an increase to 0.4 μm, two additional ultrasharp TD resonances located at 2.25 and 2.41 THz appear in the transmission spectrum under y-polarization. The sensitivity of this metamaterial in refractive index sensing can reach up to 489 GHz/RIU, demonstrating great potential for ultrasensitive sensing at terahertz frequencies^236^. Figure 8b shows a metallic QBIC metamaterial consisting of a periodic array of two ring chain resonators with different gap widths machined on a rectangular lattice^237^. Under the excitation of the THz pulse, the special up and down ring chain resonators enable strong coupling to occur in the large gap region between the resonator and the resonator cavity, effectively enlarging the sensing area. In particular, the opening width of a single square gold loop lower is variable to introduce asymmetry for achieving QBIC. It not only causes a high Q factor but also brings wide-area and intense light–matter interaction and will greatly improve the capture probability of the biosensor for trace molecules and promote the coupling of the light field energy with the sample molecules. A high sensitivity of 420 GHz/RIU is achieved, which can realize direct detection of trace molecules at the pmol level. Most of the current research focuses on optimizing the structural design to improve the performance of the metamaterial. An important but easily overlooked challenge is that the utilization rate of analytes on the device is quite limited. How to transport more analytes to enhanced hot spots is a question worth considering. A full silicon dielectric metamaterial sensor (Fig. 8c) is proposed, which excites QBIC resonance in two highly asymmetric silicon cavities, generating multiple high-Q Mie resonances in the terahertz range^238^. In the process of amino acid recognition, most analytes precipitate into the cavities, overlapping spatially with the excited hot spots, ultimately achieving qualitative and quantitative detection of three types of amino acids. It is worth mentioning that current research on terahertz sensing mainly focuses on the refractive index of analytes. However, enhancing recognizable signals by modulating resonance frequencies to match molecular characteristic absorption peaks can achieve efficient selective recognition. Lyu et al. proposed a frequency-selective fingerprint sensor, achieving narrowband α-lactose fingerprint spectrum sensing based on absorption-induced transparency, and multiplexed wideband terahertz absorption enhancement for chiral carnitine detection^239^.

Apart from the previously mentioned detection of amino acids, THz metamaterials can also be utilized for detecting cancer cells. Zhang et al. designed a metamaterial generated by two mirrored gold split ring resonators with subwavelength sizes^240^. Three types of lung cancer cells, Calu-1, A427, and 95D, have remarked differences in terms of transmittance and frequency shifts and predictable trends in the resonance dip with concentration. Their group also proposed a metamaterial biosensor composed of split ring resonators (SRRs) and cutting lines, which induced a resonant peak similar to EIT at 2.24 THz for molecular classification of glioma cells^241^. Another metamaterial with the capability of ultrafast mode switching has been reported to successfully discriminate between normal, adenoma, and cancerous states of colorectal cells, which demonstrates that metamaterial is moving forward to practical application. As shown in Fig. 8d, its core structure consists of two isolated cut wires separated by a silicon bridge and two pairs of SRRs^242^. Mode switching is achieved through controlled pump excitation combined with the silicon bridge, and with increasing pump fluences, the 1.21 THz Lorentzian lattice resonance mode gradually transitions to the 0.77 THz EIT resonance. Under the former condition, the analytes can be directly detected from the THz time-domain signal without the need for Fourier transformation. In terms of frequency domain, traditional refractive index sensing is obviously achieved under the two modes with frequency sensitivities of 96.1 and 118.4 GHz/RIU, respectively. The detection of biomolecular samples is showing an increasingly diverse trend, yet the ultimate aim of metamaterial research is to realize efficient practical applications. Addressing the demands for high sensitivity, stability, and selectivity in real-world applications is what needs to be carefully considered next.

### Combination strategy

Previously, we have described each of the four spectral enhancement techniques located in specific bands as well as two that utilize special properties. They demonstrate enormous potential for various types of molecular detection applications. From the perspective of the entire continuous spectrum, the various spectral techniques are independent of and interconnected with each other, especially the existence of ambiguous overlapping ranges between two adjacent types of light, which makes a foundation for the use of joint strategies. Besides, tunable subwavelength scale structures give metamaterials great advantages. So, they are highly desired for fabricating chip-scale sensing cores and miniaturized multifunctional sensing platforms, which set the requirements for the adoption of combination strategies.

**Fig. 9:**
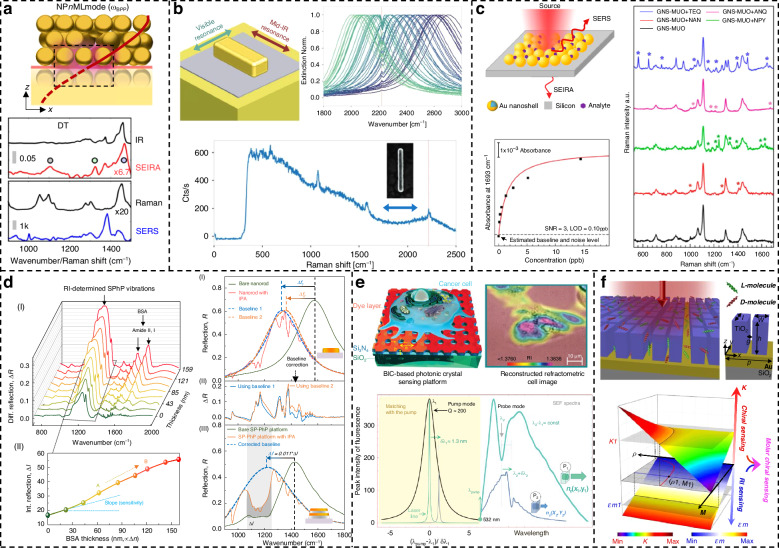
Metamaterial technology using combination strategy. **a** Mode from layers of AuNP lattice-on-mirror (generating image dipoles). SEIRA and SERS of monolayer decane-1-thiol in nanoparticle multilayer-on-mirror (seven layers) films compared to reference liquid IR/Raman spectra. Reproduced with permission^244^. Copyright 2022, Springer. **b** Schematic illustration of the NRoM construct. FTIR spectra following the assembly of a 4-MBN monolayer on the nanorods. The red-dashed line highlights the expected position of the CN vibration. Polarization-dependent SERS spectra were taken with a 772 nm laser while exciting the short axis of the NRoM. Reproduced with permission^245^. Copyright 2023, Wiley. **c** Schematic of plasmonic substrate for SEIRA and SERS composed of GNS aggregates on a Si surface. Reproduced with permission^246^. Copyright 2023, American Chemical Society. **d** Corresponding baseline-corrected molecular signals of the SP-PhP platform. (I) Two types of signals are observed, one being the RI-determined SPhP response and the other being the vibration mode of the BSA molecule (amide I, II). (II) The relationship between ΔI and BSA thickness. Comparison of baseline fitting methods. Reproduced with permission^172^. Copyright 2023, AAAS. **e** Schematic layout of the PhCS for microscopy experiments of live cells. Refractometric map reconstructed from the correlation with the spectral shift of the QBIC probe mode. Reproduced with permission^251^. Copyright 2020, American Chemical Society. **f** The values of ε_m_ and κ form two surfaces in the parameter space of ρ and M. Chiral sensing and RI sensing can respectively determine the values of ε_m_ and κ, which, in turn, give rise to two data lines in the ρ-M plane. Reproduced with permission^230^. Copyright 2020, American Chemical Society

SEIRA and SERS overlap in the near-infrared band and have been reported multiple times to achieve binding on a single substrate. Infrared and Raman spectroscopy provide complementary probes of molecular vibrations, and the development of substrates that enable the enhancement of both spectra can facilitate molecular detection for complete vibrational information. Mueller et al. show that plasmonic supercrystals are an excellent platform for enhanced spectroscopy because they possess a high density of hot spots in the electric field^243^. Through self-assembled nanoparticles, three-dimensional gold supercrystals were synthesized, which can support multiple polaritonic resonances. When testing the vibration of polystyrene molecules as spacers between the nanoparticles, the plasmon polaritons of thin supercrystals lead to SERS resonances in the near-IR, with a peak integrated enhancement of up to 300. In thicker crystals, the mid-infrared absorption of polystyrene is likewise enhanced with a maximum enhancement of 400%. Nevertheless, such highly-ordered nanoparticle superlattices face more complex regulation of fabrication and difficult access of analytes to the gap. Consequently, Arul et al. turned their attention to the hierarchical self-assembly of amorphous gold nanoparticle multilayer films on a mirror (Fig. 9a), which can possess dual resonances in the visible and infrared regions^244^. Its strong mid-infrared mode allows for both significant SEIRA and SERS enhancements up to 10^6^ enhanced factors. Structural disorder efficiently couples light into the gaps between the multilayers and mirror, enabling real sensing of sub-picolitre sample volumes. Apart from using supercrystals, nanorods-on-a-mirror (NRoM) is also a promising structure. Oksenberg et al. proposed that an optimal platform for complementary SERS and SEIRAS should provide spatially overlapping and highly enhanced fields in the VIS and mid-IR range, like the NRoM which provides sharp resonances in the VIS by short-axis excitation and mid-IR through long-axis excitation (Fig. 9b)^245^. Then they prepared a thiol self-assembled monolayer between the nanorods and the mirror substrate to detect its CN vibrations and obtained an upper bound of about 10^8^ SERS enhancement and 10^4^ SEIRA enhancement. The nanoshells (GNS), composed of SiO_2_ core and Au shell, have been shown to induce both SERS and SEIRA for molecules located within their interparticle junctions. As shown in Fig. 9c, the GNS substrate with a functionalized SAM capture layer on it can be highly useful for the specific identification and detection of non-polar polycyclic aromatic hydrocarbons (PAHs) and polar polycyclic aromatic hydrocarbons (PACs) derived with oxygen, sulphur, or nitrogen functional groups on the same substrate^246^. Nong et al. introduced Ag NPs-modified graphene nanoribbons, still capable of generating localized plasmonic resonances in the visible and mid-infrared regions, respectively, for use as shared substrates^247^. However, the enhanced properties achievable with graphene materials from applications are still quite limited.

Chiral metamaterials can be easily incorporated into SERS by preparing chiral forms of nanoparticles, examples include chiral gold nanostars^248^ and metallic nanohelices^249^ for detection. Regarding RI sensing, in addition to being widely employed in THz sensing, it holds significant promise for enhancing the precision of sensors and providing richer information within the infrared spectrum when integrated with SEIRA technology. A multiplexed dual-resonance metamaterial designed by Dong et al. achieved RI sensing in the near-infrared and ultrasensitive vibrational spectroscopy in the mid-infrared, making it more competitive than conventional metallic nanostructures^116^. However, the lack of selectivity for the medium surrounding the metamaterials leads to a rather limited contribution of RI sensing in mixed sample environments. In Fig. 9d, Zhou et al. discovered that the surface phonon polaritons (SPhP) in the polariton hybridization of their platform are sensitive to the real part of the complex RI^172^. The induced vibration change ΔI not only resolves the molecular refractive index feature but also performs high-precision joint baseline correction. Their platform achieved a 60-fold improvement in LoD, reaching physiological levels of 5 mM in glucose detection. With the help of machine learning, this strategy facilitates de-overlapping molecular fingerprints by offering supplementary features. Building on this work, Zhou et al. developed a plasmon-phonon hyperspectral imaging system using asymmetric nanoantennas^250^. By leveraging polarization-controlled phonon modes, this system captures unique refractive index features, enabling precise molecular identification. In high-speed SARS-CoV imaging assisted by deep learning, the system demonstrated enhanced sensitivity, accuracy (93%), and detection limits down to single molecular layers. In addition, utilizing the synergy between the two mechanisms of refractometric sensing and SEF has also been reported. Romano et al. demonstrate a BIC-based all-dielectric photonic crystal slab (PhCS) for cavity-enhanced hyperspectral refractometric imaging (Fig. 9e)^251^. The first resonance amplifies 2 orders of magnitude the SEF emission of a probe dye. Simultaneously, the second mode gives a Fano profile in the SEF spectrum of the dye and is tracked to correlate SEF gain and local RI variation. The minimal resolvable RI variation is estimated to be larger than 10^–5^ RIU. The similar excitation bands of SEF and SERS also offer the possibility of using the two in combination, enabling, for example, the dual-mode detection of cholesterol in serum^252^, carcinoembryonic antigen^253^, and tumor-associated miRNAs^254^. Liu et al. designed an aptamer-based biosensor for SEF and SERS detection of trace tetrodotoxin by using AuNPs-embedded MOF nanohybrids^255^. Conventional CD spectroscopy is missing information about the concentration of chiral molecules in a sample. In Fig. 9f, Chen et al. integrated the functions of chiral sensing and RI sensing into a single metamaterial, achieving molar chiral sensing^230^, which is very helpful for the detailed analysis of the enantiomeric compositions. Chiral nanospiral arrays have also been used to distinguish refractive index changes in the surrounding medium^185^. In general, the enhancement strategy using a combination of the two expands the possibilities for practical applications of metamaterials.

## Advanced integrated solutions and features

The ability of metamaterial devices to manipulate and enhance spectral signals is achieved at captivatingly small sizes, naturally providing possibilities for integration with other advanced devices and the operation of complex features. Recent advancements have highlighted the synergy between metamaterial structures and waveguide configurations, microfluidic technologies, and their applications in wearable devices. In addition, dynamic tuning technology also provides strong support for metamaterials to achieve real-time response and multifunctionality. Several works also underscore the evolving landscape where artificial intelligence plays a pivotal role, facilitating the design optimization and operational efficiency of metamaterials. Moreover, the emphasis on miniaturized modules and systems not only drives the development of compact and efficient devices but also aligns with ongoing research efforts aiming to achieve robust and portable sensing platforms across various domains.

**Fig. 10:**
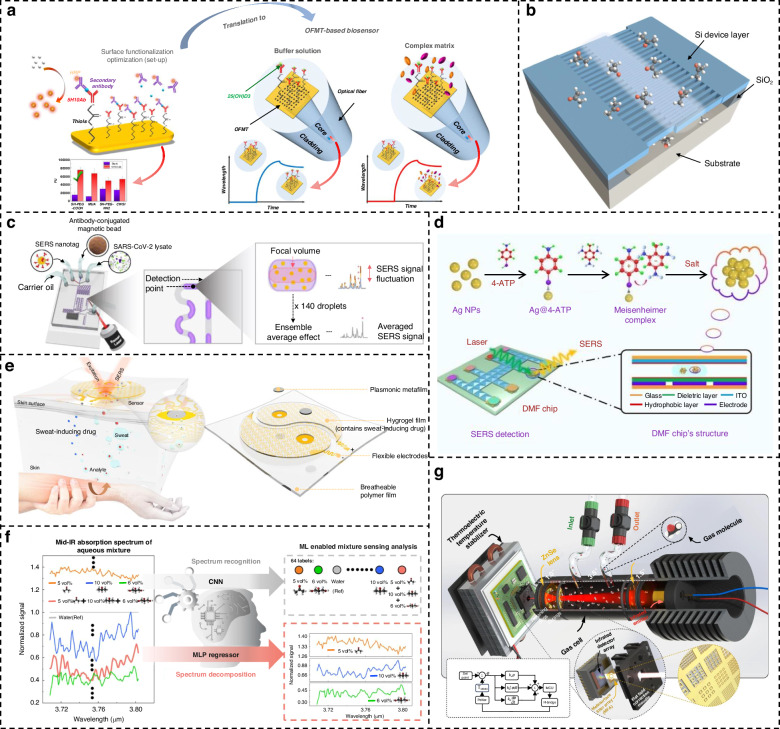
Advanced integration solutions and functions of metamaterials. **a** A lab-on-fiber biosensing platform for the detection of 25(OH)D3 based on the integration of plasmonic metamaterials on the tip of a single-mode optical fiber. Reproduced with permission^257^. Copyright 2024, Elsevier. **b** Schematic diagram of the all-dielectric metamaterial-assisted comb waveguide. Reproduced with permission^261^. Copyright 2022, American Chemical Society. **c** SERS-based microdroplet sensor for detecting SARS-CoV-2 lysate. Reproduced with permission^264^. Copyright 2022, Elsevier. **d** Schematic illustration of the SERS-based detection of trace explosives combined with digital microfluidics. Reproduced with permission^267^. Copyright 2023, American Chemical Society. **e** Schematic drawing showing the working principle and design of the device. Reproduced with permission^275^. Copyright 2021, AAAS. **f** Machine learning for two sensing functions: the CNN is for recognizing concentration combinations, while the MLP regressor is used for mixture spectrum decomposition. Reproduced with permission^298^. Copyright 2022, American Chemical Society. **g** Schematic of metamaterial gas sensing system. Reproduced with permission^306^. Copyright 2024, Springer

Although waveguides are widely utilized in electromagnetic communication, integrating a metamaterial structure with a waveguide platform enhances spectral capabilities, facilitating more versatile applications. This integration allows for efficient light guidance and significant interactions with the surrounding environment, which also implies the possibility of building fully integrated on-chip spectral surface enhancement systems. With advances in manufacturing processes, a prevalent configuration involves incorporating a metamaterial at the tip of an optical fiber, thereby creating a lab-on-tip platform. Consales et al. reported a novel biosensing platform based on the integration of a phase-gradient plasmonic metamaterial on the tip of an optical fiber, able to detect biomolecular interactions with very high sensitivity^256^. They demonstrated that the proposed platform can detect very low concentrations of streptavidin in running buffer solutions, with a LOD of a few ng/mL. Then, Cusano et al. developed a similar Optical Fiber Meta-Tip for the detection of vitamin D in both saline buffer and in a complex matrix^257^. Figure 10a illustrates their biosensing platform. A dedicated pipeline was carefully designed and developed to optimize the bio-functionalization of the plasmonic sensor tip to specifically detect the target biomolecule. These lab-on-tip configurations are poised to integrate with medical catheters for liquid biopsy applications in real-time medical diagnostics. In addition to optical fibers, metamaterials are often integrated with dielectric waveguides and plasmon waveguides, for example, gold nanorods and coaxial nanoapertures which built atop a silicon-on-insulator waveguide platform^258^, and the SPP waveguide which is fabricated with a thin dielectric stripe on top of an unpatterned gold surface^259^. Chen and colleagues showcased ultra-compact plasmonic resonators directly fabricated on a silicon waveguide, enabling mid-infrared spectroscopic chemical sensing applications^260^. Their plasmonic nanorod resonators occupy a footprint as diminutive as 2 μm^2^. The low-loss nature of all-dielectric metamaterials is equally compelling for coupling with optical waveguides. As shown in Fig.10b, Liu et al. proposed an ultrasensitive all-dielectric metamaterial-assisted comb waveguide sensor featuring a larger than-unity external confinement factor as well as a low propagation loss^261^. Acetone absorption spectroscopy is demonstrated using their sensor around 7.33 μm, showing a detection limit of 2.5 ppm with a waveguide length of only 10 mm. Typically, it has been assumed that dielectric metamaterial structures provide fundamentally worse electromagnetic field confinement than metallic structures, but recently, Lepeshov et al. ^262^ demonstrated that light can be highly localized in dielectric periodic structures by exploiting the vanishing fundamental Fourier harmonic of the Bloch eigenmode. This mechanism can be used to improve optical sensors based on all-dielectric metamaterials by increasing the overlap between optical modes and thin layers of bio-analytes.

Microfluidic chips integrate multiple functional units on a single platform to precisely manipulate minute volumes of fluids and conduct comprehensive sample analysis processes. In contrast to more established liquid pumping and control units, the analytical units often rely on external bulky equipment. The small scale of nanoparticles and metamaterials demonstrated high adaptability with microfluidic chips from the outset, significantly enhancing efficient handling and sensitive detection capabilities of fluid samples. This integration sets the stage for the realization of fully autonomous Lab-on-Chip systems. SERS is widely integrated into microfluidic systems due to the small effect of water molecules on the Raman scattering signal of the target material. Jeon et al. proposed a SERS-based droplet gradient chip composed of two layers^263^. One layer is a panel for serial dilution of a reagent, and the other is a panel for gold nanoflowers distribution and droplet generation. This fully integrated SERS-based microdroplet platform is expected to be a powerful analytical tool for high throughput detection of a chemical or biological reagent. As shown in Fig. 10c, Park et al. developed a SERS-based microdroplet sensor for rapid, sensitive, and reproducible SARS-CoV-2 detection^264^. In particular, the Raman signal was measured across multiple microdroplets containing SERS nanotags, significantly enhancing the detection sensitivity of this sensor through ensemble averaging. This method successfully detected SARS-CoV-2 at a low concentration of 0.22 PFU/mL with a coefficient of variation of 1.79%. Zhou et al. utilizes a microfluidic platform integrated with surface-enhanced Raman scattering for multiplex profiling of sEV glycans in non-small cell lung cancer^265^. The results successfully differentiate patients with early-stage malignant lung nodules from benign lung nodules. Traditional SERS detection is based on single-focus excitation scenarios, Dong et al. proposed a SERS microfluidic chip with a barium titanate microspheres array embedded for SERS detection with simultaneous enhancement of sensitivity and stability^266^. Emerging digital microfluidics (DMF) enables droplet generation, movement, mixing, and splitting, and its integration allows for better automation of sample detection. Figure 10d illustrates a SERS-DMF platform for automated, high-throughput, and high-sensitivity detection of explosives by Liu et al. ^267^. Different concentrations of target molecules, silver nanoparticles, and salts were loaded into the DMF chip to generate hot spots. The results showed that the detection limits for TNT and NTO using this platform were 10^−7^ and 10^−8^ M, respectively. Das et al. reported a novel approach for SERS detection in DMF by a microspray hole that uses an electrostatic spray for sample transfer from inside the chip to an external SERS substrate^268^. Despite the unparalleled advantages of integrating SERS with microfluidics, it is essential to consider fully leveraging the information from each remaining spectroscopic technique. Kavungal et al. developed an immunoassay-coupled nanoplasmonic infrared metamaterial sensor that detects proteins linked to neurodegenerative disorders^269^. By integrating with microfluidics, their sensor can retrieve time-resolved absorbance fingerprints in the presence of a complex biomatrix and is capable of multiplexing for the simultaneous monitoring of multiple pathology-associated biomarkers. A recent study found that characteristic features due to Fresnel reflections at different dielectric interfaces of the plasmonic sensors, including a microfluidic chamber, help improve sensing performance^270^. Unlike mere surface-level integration of metamaterials on microfluidic chips, this approach considers their mutual interactions, indicating that by optimizing the integration scheme, sensor performance can be further enhanced beyond the existing capabilities.

Wearable sensing technologies enable non-invasive sampling and real-time monitoring of the human body, representing a critical direction for future advancements in medical care. Utilizing flexible materials as substrates for metamaterials^271,272^ offers a pathway toward implementing various sensitive wearable sensing applications. Natural attention is drawn to the analysis of human sweat composition. He et al. demonstrated a wearable microfluidic nanoplasmonic sensor capable of refreshable and portable recognition fingerprint information of targeted biomarkers, including urea, lactate, and pH in sweat^273^. The sensor collects sweat through a microfluidic chip and flows into a silver nano mushroom array in an intermediate chamber isolated from the skin, avoiding various risks of using a permeable SERS substrate. The fingerprint information is then decoded in real-time by a portable Raman spectrometer. Yang et al. developed a more durable wearable plasma paper-based microfluidic device under a similar architecture^274^. Also using SERS technology, the flexible SERS wearable metamaterial shown in Fig. 10e successfully detects trace changes in various drugs within the human body^275^. Beyond wearable sensors for human applications, monitoring the growth process and health status of plants is also an increasingly prominent area of interest^276^. Unfortunately, existing reports are still dominated by electrochemical-based technologies^277^. As of now, the application of flexible plasmonic metamaterials in wearable devices is relatively limited. Metamaterial sensors typically require the fabrication of fine and uniform periodic structures on rigid substrates to ensure exceptional performance, which poses challenges for adapting to wearable applications. The proper utilization of flexible materials and the impact of deformation on metamaterial sensing still warrant further exploration. Furthermore, the size of the spectrometers used for subsequent measurements significantly limits their feasibility, necessitating the development of versatile portable spectrometers to enable the applicability of numerous spectroscopic technologies beyond SERS.

Tunable metamaterials overcome the inherent limitations of traditional static metamaterials, opening vast possibilities for precise wavefront manipulation and reconfigurable metamaterials^278^. With the rapid development of metamaterials, the limitations of static metamaterials in practical applications—such as narrow bandwidth and lack of flexibility in wave manipulation—have become increasingly prominent^279^. Consequently, researchers have devoted significant efforts to the study of tunable metamaterials. Currently, the common tuning mechanisms for metamaterials include electrical^278^ and thermal control^280^, which typically rely on specially designed active materials. The optical properties of these active materials can be modulated by external stimuli such as pressure, heat, electric current, and light^281^. By integrating these active materials into metamaterials, the interaction between light and matter can be dynamically controlled under external stimuli. This external stimuli-driven tunable metamaterial approach provides greater freedom for dynamically manipulating and controlling electromagnetic waves. Common active materials include phase-change materials (PCMs), transparent conductive oxides (TCOs), liquid crystals (LCs), and atomically thin materials^281^. These materials exhibit fascinating properties such as fast switching speeds, large modulation depths, ultracompactness, and significant optical contrast under external stimulation. For example, Aigner et al. proposed an active metasurface approach based on phase-change materials^282^. By combining the temperature-tunable losses of vanadium dioxide (VO₂) with symmetry-protected BIC for tunable far-field coupling, this approach achieves precise and independent control of radiative and nonradiative losses, enabling switching between different coupled states in metamaterials. Beyond the aforementioned tuning mechanisms, mechanical tuning methods for metamaterials have also been extensively explored. Due to the typically small size of metamaterials, mechanical control often involves integrating metamaterials with microelectromechanical systems (MEMS)^283–285^. Typical MEMS metamaterials utilize various actuation methods, including electrostatic actuators (ESAs)^286^, electrothermal actuators (ETAs)^287^, and electromagnetic actuators (EMAs)^288^. Among these, ESA is one of the most attractive methods for MEMS-based metamaterials due to its compact device design, repeatable mechanical deformation, and low power consumption. Additionally, fabricating metamaterials on flexible substrates enables the creation of novel stretchable metamaterials^289^. In recent years, the combination of microfluidic systems with metamaterials has introduced a new platform for actively controlling metamaterials, paving the way for electromagnetic wave manipulation and advanced devices^290,291^. Notably, the liquid background in such fluid metamaterials can be modulated by adding chemical reagents or changing the liquid composition, thereby changing the refractive index or conductivity of the liquid and, ultimately, the optical response of the metamaterial^292^. The unique tunable properties and flexibility of fluidic metamaterials present exciting possibilities for various applications, including material sensing, biological detection, and imaging. For instance, Song et al. demonstrated a metamaterial based on liquid metal using microfluidic technology^293^. The liquid metal pillar array can be continuously controlled vertically through microfluidics. Experimental results showed that the absorption frequency of the proposed terahertz metamaterial could be efficiently tuned within the range of 0.246 to 0.415 THz, achieving a central frequency tuning range of 51.1%.

The maturity of artificial intelligence has catalyzed significant advancements across various scientific disciplines, with the wave of machine learning extending into photonics, plasmonics, and metamaterial domains, showcasing notable achievements in structural design and sensor data processing. On the one hand, deep learning techniques can be applied to the inverse design of metamaterial structures, emphasizing precise implementation of requirements while bypassing complex design processes. Qiu et al. connected the metamaterial structure with its electromagnetic properties through the deep learning method, where they set up and trained the model with a set of samples^294^. Once a design target of the metamaterial is input into a trained deep-learning model, the structure of the metamaterial will be generated automatically. Using this method, they designed a triple-band metamaterial absorber, and the structure performs in good accordance with the design target. Kanmaz et al. proposed the encoder-decoder neural network, showing high performance for both the forward and inverse design problems^295^. However, networks trained in a conventional manner exhibit specificity and cannot connect different types of metamaterials. Hence, the concept of the inheritance paradigm emerges, which means that neural networks can inherit the knowledge from the “parent generation” and then become freely assembled to construct an “offspring” neural network^296^. Such a framework is poised to curtail data requirements and augment network flexibility^297^. On the other hand, machine learning and its subset deep learning have shown remarkable effectiveness in prediction and clustering. The classical algorithms often used in the multicomponent analysis of optical sensors are principal component analysis (PCA), support vector machines (SVM), deep neural networks (DNN), etc. For example, in combination with DNN algorithms, the RI-determined SPhP vibrations can decouple overlapping IR vibrational modes. In the demonstration of dynamic profiling glucose enzymatic reaction, all bond-breaking-bond-making events were observed with a 92% identification accuracy, even for strongly overlapping vibration modes^172^. Also, Aided by a DNN based on a multilayer perceptron (MLP) model, the near-field enhanced amide II and I absorption signals of an ImmunoSEIRA sensor quantitatively identify the percentage presence of aSyn oligomers and fibrils in their mixture^269^. In a metamaterial waveguide sensing platform for MIR aqueous mixture analysis presented by Zhou et al. (Fig. 10f), the convolution neural network (CNN) is employed to recognize the absorption spectra of mixtures with 64 predefined mixing ratios, and a classification accuracy of 98.88% is achieved^298^. Additionally, they executed the MLP regressor on the 64 mixture spectra for spectrum decomposition and concentration prediction. Recently, Liu et al. presented an AI-enhanced waveguide photonic nose for VOC gas mixture analysis in MIR as an augmented sensing platform, again demonstrating the ability of AI-assisted facilitated sensing and analysis^299^.

The previous content has presented a lot of integration schemes for metamaterial structures, demonstrating the richness of functionality that can be achieved in combination with specialized equipment, and advanced technologies. Artificial intelligence was mentioned as a valuable tool in guiding metamaterial design and post-sensing processing. In this paragraph, we emphasize the characteristic of miniaturization, which serves as both the foundation for developing numerous integrated solutions and the ultimate goal tirelessly pursued by researchers. The existing optical detection systems are usually large desktop instruments composed of bulky components, which greatly limit their usage scenarios. The demand for portable spectral detection devices is rapidly increasing across various fields such as agriculture, environment, and healthcare. Obtaining instant and effective on-site results without the need to transport samples to a laboratory is highly appealing, as it significantly reduces the time and spatial costs of spectral detection. As the size of the systems further miniaturizes to the centimeter or even sub-millimeter scale, their application scope expands further. They can be integrated as functional components into various mobile portable devices. At this point, the pursuit of high performance can also yield to the demand for miniaturization^300^. Firstly, in terms of the specific need for the identification of narrowband spectra, optical gas sensors have had a breakthrough in compact size and energy efficiency with little sacrifice in sensitivity and robustness. Lochbaum et al. proposed a design using a metamaterial thermal emitter and a corresponding metamaterial perfect absorber (MPA), which has an integrated thermopile as the receiving end of the sensor. Combined with a non-resonant cavity measured only 5.7 × 5.7 × 4.5 mm^3^, the device is shown to match the narrow absorption band of CO_2_ gas, with a sensitivity of 22.4 ± 0.5 ppm/Hz^0.5^ ^301^. Furthermore, many researchers are paying close attention to more universally applicable micro-spectrometers in the field of detection. Currently, the development of the detector part of a miniature spectrometer is quite mature, and reliable choices are the CMOS image sensor commonly found in smartphones^302^ and infrared detector arrays for thermal imaging applications. Using metamaterial structures as a filter array provides a crucial advantage for achieving miniaturized spectral detection by combining compactness and sensitivity. Combined with commercially available CMOS image sensors, Jian et al. demonstrated a silicon real-time ultraspectral imaging chip based on reconfigurable metamaterials for imaging brain hemodynamics, and the dynamic spectral absorption properties of deoxyhemoglobin and oxyhemoglobin in a rat barrel cortex were obtained^303^. Tittl et al. prepared arrays of dielectric metamaterials forming a series of discrete sharp resonances for the detection of mid-infrared molecular fingerprints, proposing the idea of a miniaturized and integrated solution using an external broad light source, combined with an infrared imaging detector^169^. Then, Meng et al. combined a plasma metamaterials spectral filter and an uncooled thermal imager into a mid-infrared filter array-detector-array (FDAD) spectrometer, in which rectangular aluminum rings on a silicon substrate and rectangular aperture metamaterials in an aluminum film were designed to achieve band-resistance filtration and band-pass filtration, respectively. The output of the system was analyzed by machine learning algorithms to achieve classification and concentration detection using simulated noisy information on a wide range of gas- and liquid-phase chemicals^304^. Later, they further considered the processing of the interpreted output spectra on the basis of the previously modeled structures and prepared a MIR microspectrometer with a volume of only about 1 cm^3^ to experimentally demonstrate the tasks of chemical sensing and quantification. Their system was highly accurate in a group of liquids analyte identification, predicting the concentration of specific liquid samples at dilutions as low as 0.1%, in addition to demonstrating the ability to classify pharmaceuticals and edible oils^305^. Figure 10g shows their latest metamaterial microspectrometer gas sensing system^306^. In a word, the advancements in the miniaturization of optical sensors and micro-spectrometers are revolutionizing spectral detection technologies. These achievements are paving the way for more compact, energy-efficient, and universally applicable devices and constructing a new detection system pattern.

## Conclusions and outlook

As researchers make systematic progress on metamaterials, enhanced spectroscopy will grow rapidly in the future and show promising applications in several fields. The focus of metamaterial research has eventually shifted from new principles, new phenomena, and new structures to new properties and new functions for technological applications^307^. Specifically, it may be expanded and deepened in the following directions: (i) Tunability: Future metamaterials will be designed to be more tunable, with the ability to be adjusted and optimized in different wavelength ranges as required. It is expected that the tunability can be realized at the use stage of the product rather than only in the design process, thus making the technology more widely applicable and flexible in different application scenarios. (ii) Miniaturization: As discussed in detail in the section “Advanced integrated solutions and features”, miniaturization will be one of the important development directions for future metamaterials enhanced spectroscopy techniques. Through micro and nanofabrication techniques, miniaturized optical components and structures can be realized, thus reducing the size and weight of the equipment. Such miniaturized systems will be more suitable for portable and real-time monitoring applications, while also helping to save resources and improve energy efficiency. (iii) Integration: With the development of micro- and nanofabrication technologies, metamaterials can be combined with other units to achieve integration of the entire spectroscopy system for more convenient and efficient spectroscopy and detection. Integration will improve the stability and reliability of the system, help to reduce costs, and simplify the operation process. (iv) Multifunctionality: Based on miniaturization and integration, it is expected that a variety of spectral analysis or sensing functions can be realized on a single platform, even detection functions other than spectral detection technology. Multifunctionality will improve the technology’s wide range of applications and economic benefits. (v) Intelligent: Future metamaterials may have adaptive and intelligent detection capabilities, combined with advanced machine learning, artificial intelligence, and other processing algorithms to provide highly sensitive and stable performance.

In summary, the review presents a complete and detailed overview of six metamaterials-based surface-enhanced spectroscopy techniques, including SEF, SERS, SEIRA, THz sensing, RI sensing, and chiral sensing, from the entire spectral range. Starting from the generation of optical resonance mechanisms and the principles of spectral enhancement, the review delves into the recent new developments of the six techniques in the field of sensing, complemented by the use of joint strategies. We particularly emphasize its enormous potential for developing miniaturized high-performance devices, explore recent advanced integration solutions and functionalities, and conclude with a discussion of future directions. It is foreseen that the progress in the maturation of surface-enhanced spectroscopic techniques is depicting a bright future.
